# The Longitudinal Relationship Between Self-Reported Executive Function and Mental Health in Early Adolescence

**DOI:** 10.1016/j.jaacop.2025.07.003

**Published:** 2025-08-06

**Authors:** Verena Hinze, Sarah-Jayne Blakemore, Tim Dalgleish, Tamsin Ford, Karen L. Mansfield, Obioha C. Ukoumunne, Willem Kuyken, Jesus Montero-Marin

**Affiliations:** a University of Oxford, Oxford, United Kingdom; b University of Cambridge, Cambridge, United Kingdom; c University of Exeter, Exeter, United Kingdom; d Parc Sanitari Sant Joan de Déu, Sant Boi de Llobregat, Spain; e Consortium for Biomedical Research in Epidemiology & Public Health (CIBER Epidemiology and Public Health—CIBERESP), Madrid, Spain

**Keywords:** adolescence, executive function, mental health, suicidality, well-being

## Abstract

**Objective:**

Psychological theories emphasize the role of executive function in the mental health of adolescents. Yet, the longitudinal relationship remains poorly understood. This cohort study explored the longitudinal relationship between self-reported executive function and adolescents’ mental health and potential gender differences.

**Method:**

Data were collected at 3 time points from 8,072 adolescents (11-15 years old) in 84 secondary schools in the United Kingdom, as part of the MYRIAD (MY Resilience In ADolescence) trial (ISRCTN86619085). The longitudinal relationship between adolescents’ self-reported executive function (Behavior Rating Inventory of Executive Function, Second Edition [BRIEF-2]) and 4 mental health outcomes—well-being (Warwick-Edinburgh Mental Wellbeing Scale [WEMWBS]), social-emotional-behavioral difficulties (Strength and Difficulties Questionnaire [SDQ]), risk for depression (Center for Epidemiologic Studies Depression Scale [CES-D]), and suicidality (item-based)—was explored using 3 mixed-effects regression models. Model 1 included a composite measure of executive function; model 2 included behavioral, cognitive, and emotional self-regulation; and model 3 included 7 executive function skills.

**Results:**

Better executive function (ie, lower BRIEF-2 scores) was associated with better mental health over 1 year (regression coefficient [95% CI]: well-being −0.23 [−0.24, −0.22], social-emotional-behavioral difficulties 0.24 [0.23, 0.24], risk for depression 0.33 [0.32, 0.34], and suicidality 0.02 [0.01, 0.02]). This association weakened over time for all outcomes except suicidality (model 1). Associations were strongest for emotional self-regulation (model 2) and specifically emotional control and planning (model 3). The relative role of other executive function skills (eg, working memory and self-monitoring) differed by outcome and gender.

**Conclusion:**

Better executive function was associated with better mental health over time. Potential intervention targets include emotional self-regulation, particularly emotional control and planning.

## Plain language summary

This study used data from 8,072 adolescents (aged 11-15 years) in 84 United Kingdom schools, collected as part of the MYRIAD (MY Resilience In ADolescence) trial, to examine how executive function relates to adolescent mental health outcomes over one year. Young people who reported better executive function were more likely to report better mental well-being, as well as less social-emotional-behavioral difficulties, depression, and suicidality, over time. The relation to mental health outcomes at one year was strongest for better emotional self-regulation, including emotional control and planning, reported at baseline in both boys and girls.

Mental health difficulties typically emerge before adulthood, with the earliest peak in incidence at age 15, though many emerge earlier.^1^^,^^2^ Early onset, especially in childhood and adolescence, is associated with longer symptom duration and higher rates of mental health comorbidities.^3^ The term adolescence refers to a distinct developmental period between the ages of 10 and 19 years.^4^ This period involves significant physical, cognitive, emotional, and social changes that may impact long-term mental health outcomes.^5^ As growing evidence highlights deteriorating mental health in adolescents, particularly in adolescent girls,^6^, ^7^, ^8^ understanding modifiable factors that affect mental health outcomes during this crucial developmental period is paramount to inform effective intervention, prevention, and mental health promotion strategies.^9^

Psychological theories emphasize the role of executive function in mental health of adolescents.^10^ Executive function refers to a set of cognitive capacities that enable goal-directed behavior, including self-regulation of one’s thoughts, emotions, and behavior.^11^ These self-regulation skills are enabled by specific abilities, including set-shifting (to flexibly switch between tasks or mental sets), working memory (to mentally hold and manipulate multiple pieces of information at once), and inhibitory control (to choose one’s attentional focus, cognitions, behavior, and emotions by suppressing competing internal impulses or external distractions to pursue future goals). Other skills include self-monitoring (to observe and evaluate one’s own thoughts, emotions, and behavior), emotional control (to regulate and modulate one’s emotional responses to adapt to situational demands), task completion (to sustain attention, resist distraction, and persist with a task until it is completed), and planning (to generate and organize a series of steps to accomplish one’s goals).^10^^,^^12^^,^^13^ These skills typically improve with age, allowing young people to flexibly adapt to the changing circumstances in adolescence.^14^ However, stressful life events and early childhood trauma can hinder the development of executive function skills.^15^

This is important, as executive function is involved in multiple aspects of daily life, ranging from mental and physical health to social relationships and academic/work performance.^10^^,^^16^ A recent meta-analysis of 167 longitudinal studies found that greater executive function during childhood and adolescence is associated with a reduced risk of future externalizing problems (eg, social and behavioral difficulties) and depressive symptoms (but not anxiety symptoms), suggesting that executive function may be an important transdiagnostic treatment target for a range of mental health difficulties in both clinical and community-based samples.^14^ However, it remains unclear whether these associations with specific mental health outcomes are driven by executive function in general or by specific executive function skills.^14^ Recent research suggests that higher emotional reactivity in childhood is associated with an increasing probability of internalizing difficulties in early adolescence.^17^ In the absence of exploring distinct mental health outcomes, little is known about the predictive value of executive function, its subdimensions and skills, in relation to specific mental health outcomes over time. Furthermore, although some studies suggest gender differences in specific executive function skills (eg, improved working memory in women), the evidence is mixed, and gender differences may depend on different strategies used in behavioral tasks.^18^ Indeed, previous research suggests that differences in emotion regulation strategies (eg, rumination in women and substance use in men) may explain gender differences in mental health difficulties (eg, higher rates of depression and anxiety in women and substance use difficulties in men).^19^ More rigorous research is needed to understand how specific executive function skills may affect different mental health outcomes in boys and girls over time.

As executive function skills are amenable to change, identifying specific skills that can be trained might offer an opportunity to improve mental health outcomes in early adolescence.^20^^,^^21^ The MYRIAD (MY Resilience In ADolescence) trial^22^^,^^23^ compared school-based mindfulness training with usual social-emotional teaching and found no improvements in self-reported executive function or mental health outcomes, primarily due to a lack of student engagement with the mindfulness practice.^24^, ^25^, ^26^ Previous research showed that, on average, students engaged with the practice once during the intervention period (range 0-5 times).^26^ In the absence of intervention effects, this trial enabled an exploration of the longitudinal relationship between self-reported executive function and mental health outcomes in early adolescence to inform effective intervention and prevention strategies during this critical stage of life.

This study used MYRIAD trial data to explore the longitudinal relationship between self-reported executive function (and underpinning skills) and mental health outcomes, ranging from mental well-being to suicidality, in early/middle adolescence. We explored whether these relationships were stable or changed over time and potential gender differences.

## Method

### Design and Participants

This secondary data analysis uses MYRIAD trial data (ISRCTN86619085).^22^ Informed assent/consent was obtained from schools, parents (opt-out), and adolescents. Adolescents were eligible if they provided informed assent and understood English. Previous work showed that trial schools and students were representative of secondary schools and students in the United Kingdom.^24^

**Figure 1:**
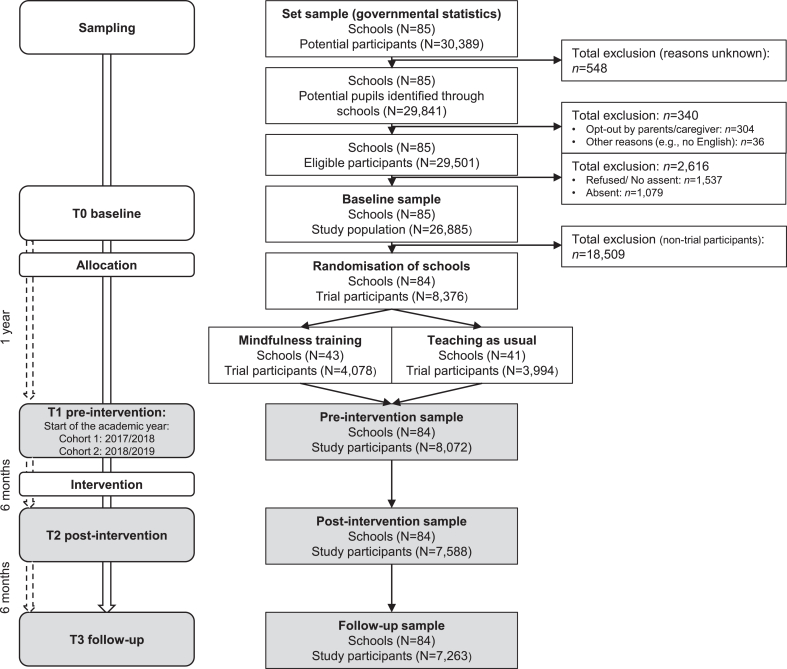
Flow Diagram

Data were collected from 8,072 adolescents in 84 UK secondary schools at 3 time points, each separated by 6 months (T1-T3) (Figure 1), starting in the academic years 2017/2018 (cohort 1: n = 923) and 2018/2019 (cohort 2: n = 7,149) and concluding before the COVID-19 pandemic. As school-based mindfulness training (vs usual social-emotional learning) did not improve executive function and mental health outcomes in adolescence,^24^ primarily due to a lack of engagement with the mindfulness practice,^26^ we analyzed participants from both trial arms together and adjusted for trial arm allocation (ie, school-based mindfulness training vs usual social-emotional learning) by including this variable as a covariate in our models.

Ethical approval was obtained from the University of Oxford Central University Research Ethics Committee (R45358/RE001; 23/05/2016). For further details, see the protocol and update.^22^^,^^23^

### Measures

Adolescents completed self-report questionnaires on their mental well-being (Warwick-Edinburgh Mental Wellbeing Scale [WEMWBS]; score range 14-70),^27^^,^^28^ social-emotional-behavioral difficulties (Strength and Difficulties Questionnaire [SDQ]; score range 0-40),^29^^,^^30^ risk for depression (hereafter referred to as depression) (Center for Epidemiologic Studies Depression Scale [CES-D]; score range 0-60),^31^^,^^32^ and suicidality (using 3 standardized questions that were specifically developed for this study).^22^ We combined the suicidal and self-harm questions into 1 variable (hereafter referred to as suicidality), reflecting increasing severity (0 = low risk; 1 = life was not worth living; 2 = self-harm thoughts; 3 = self-harm behaviors) (Supplement 1, available online).^33^

**Figure 2:**
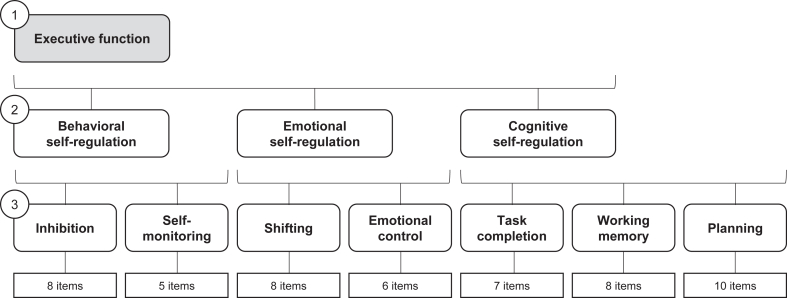
Executive Function Measurement Structure ***Note:****This figure shows the 3-level factor structure of the Behavior Rating Inventory of Executive Function, Second Edition,**^34^**which we replicated using confirmatory factor analysis (comparative fit index = 0.900; Tucker-Lewis index = 0.895; root mean square error of approximation [90% CI] = 0.039 [0.039-0.040], standardized root mean squared error = 0.042). The different possible score ranges for the subscales and skills are as follows: executive function, 52-156; behavioral self-regulation, 13-39; emotional self-regulation, 14-42; cognitive self-regulation, 25-75; inhibition, 8-24; self-monitoring, 5-15; shifting, 8-24; emotional control, 6-18; task completion, 7-21; working memory, 8-24; planning, 10-30. Higher scores mean greater difficulties.*

Self-reported executive function and subdimensions/skills were measured with the Behavior Rating Inventory of Executive Function, Second Edition (BRIEF-2).^34^ Figure 2 summarizes the factor structure, which we replicated using confirmatory factor analysis. This questionnaire provides a composite measure of self-reported executive function (raw score range 52-156), underpinned by behavioral, emotional, and cognitive self-regulation and 7 executive function skills (inhibition, self-monitoring, shifting, emotional control, working memory, task completion, and planning). Higher scores reflect greater self-reported difficulties. Consistent with previous reports and the official scoring manual,^34^^,^^35^ the BRIEF-2 demonstrated good to excellent reliability (internal consistencies 0.79-0.97) with moderate to strong validity (item–pair correlations 0.41-0.63, inconsistency score 99.3% acceptable), supporting its suitability as a self-report measure of executive function in adolescents (Supplement 1, available online). We explored executive function and its subdimensions separately to obtain a more detailed understanding of the relationship between executive function and mental health outcomes in adolescence to identify potential intervention targets. Demographic characteristics included adolescent’s age, gender (male, female, other/prefer not to say), and ethnicity (White British, Asian, Black, mixed/other ethnic minorities) (see also Supplement 1).

### Statistical Analyses

Participant characteristics are described using means (SDs) and counts (percentages). We undertook complete case analyses and explored missingness. We explored the longitudinal relationships between self-reported executive function (including behavioral, emotional, and cognitive self-regulation and 7 executive function skills) and 4 mental health outcomes—mental well-being, social-emotional-behavioral difficulties, depression, and suicidality. To explore longitudinal rather than repeated cross-sectional relationships, we used time-constant predictors measured at T1 and time-varying outcomes measured at T1 through T3. Time was categorical (coded as T1 = 1 [reference]; T2 = 1.5; and T3 = 2, to reflect the 6-month interval between the assessments). We fitted 3-level random intercept linear regression models using maximum likelihood estimation (level 1 = repeated measurements; level 2 = students; level 3 = schools). All predictors were centered at the school level to show the predicted change in adolescents’ mental health outcomes associated with within-school changes in the predictors. Convergence issues due to multicollinearity were addressed by using a quadratic approximation. We used 2-sided contrasts with .05 significance level and interpreted 95% CIs.

We examined self-reported executive function 1 measure at a time (composite score, 3 self-regulation dimensions, and 7 skills). To determine whether the association between each predictor (at T1) and mental health outcome (T1-T3) changed (strengthened/weakened) or remained stable over time, we compared models with a time × predictor interaction with models with only the main effects. A significant improvement in fit for the interaction model over the main effects model (likelihood ratio test *p* < .05) indicated changes in the longitudinal association. Regression coefficients for the time × predictor interaction reflect changes in the longitudinal relationship between self-reported executive function predictors (at T1) and adolescents’ mental health outcomes over time relative to the first assessment (T1). A stable longitudinal relationship was inferred if the model with the interaction term did not enhance fit (likelihood ratio test *p* > .05). Here, regression coefficients show the average relationship over time.

To assess the unique contribution of each predictor on adolescents’ mental health outcomes over time, we included statistically significant main or time × predictor interaction terms in 3 multivariable models: model 1 (composite score), model 2 (3 self-regulation dimensions), and model 3 (7 executive function skills). This approach allowed us to compare our findings with the literature that has predominantly focused on composite executive function measures or emotional self-regulation and to identify specific skills as potential intervention targets. We included cohort, allocation, age, and ethnicity as potential covariates in the multivariable models. Furthermore, we corrected *p* values for multiple comparisons using the Benjamini-Hochberg method.^36^

To explore gender differences, we conducted separate analyses for girls and boys to simplify models (by avoiding 3-way interactions) and facilitate future meta-analyses through a direct comparison of regression coefficients. Non-overlapping 95% CIs suggested gender differences in the respective effect. Analyses were undertaken in R version 3.6.2 (R Foundation for Statistical Computing, Vienna, Austria) (Supplement 2, available online).

## Results

Our sample consisted of 8,072 adolescents (84 schools) with mean age of 13 years (range 11-15 years; 93% aged 12-13 years), including 55% female, 43% male, and 2% other/prefer not to say (Table 1). See Table S1, available online, for a descriptive comparison of participant characteristics at T1 by gender. The findings indicate slightly higher mean levels of mental health difficulties and poorer emotional self-regulation at T1 among girls and particularly nonbinary youth (other/prefer not to say) compared with boys. Given the small numbers of nonbinary youths, separate analyses were conducted for girls and boys only.

**Table 1:** Participant Characteristics Across Time Points

	T1 (k = 84 schools; N = 8,072)				T2 (k = 84 schools; n = 7,588)				T3 (k = 84 schools; n = 7,263)			
			Missing n	(%)			Missing n	(%)			Missing n	(%)
	**n**	**(%)**			**n**	**(%)**			**n**	**(%)**		
Cohort			0	(0)			0	(0)			0	(0)
1	923	(11.4)			871	(11.5)			867	(11.9)		
2	7,149	(88.6)			6,717	(88.5)			6,396	(88.1)		
	**Mean**	**(range)**			**Mean**	**(range)**			**Mean**	**(range)**		
Age, y	13.1	(11.9-15.2)	0	(0)	13.7	(12.6-15.8)	0	(0)	14.1	(13.0-16.3)	0	(0)
Gender			145	(1.8)			134	(1.8)			131	(1.8)
	**n**	**(%)**			**n**	**(%)**			**n**	**(%)**		
Female	4,380	(55.3)			4,126	(55.4)			3,953	(55.4)		
Male	3,389	(42.8)			3,181	(42.7)			3,044	(42.7)		
Other/prefer not to say	158	(2.0)			147	(2.0)			135	(1.9)		
Ethnicity			172	(2.1)			159	(2.1)			153	(2.1)
Asian	819	(10.4)			785	(10.6)			762	(10.7)		
Black	407	(5.2)			365	(4.9)			345	(4.9)		
Mixed and other ethnic minorities	707	(8.9)			648	(8.7)			619	(8.7)		
White British	5,967	(75.5)			5,631	(75.8)			5,384	(75.7)		
	**Mean**	**(SD)**			**Mean**	**(SD)**			**Mean**	**(SD)**		
WEMWBS score	49.1	(9.1)	14	(0.2)	47.9	(9.5)	16	(0.2)	47.6	(9.8)	19	(0.3)
Probable mental health difficulties	1,371	(17.0)			1,566	(20.7)			1,619	(22.3)		
Possible mental health difficulties	967	(12.0)			1,026	(13.5)			981	(13.5)		
Average mental well-being	4,762	(59.1)			4,219	(55.7)			3,907	(53.9)		
High well-being	958	(11.9)			761	(10.1)			737	(10.2)		
	**Mean**	**(SD)**			**Mean**	**(SD)**			**Mean**	**(SD)**		
SDQ score	12.4	(6.6)	30	(0.4)	13.3	(6.9)	46	(0.6)	13.1	(6.8)	38	(0.5)
	**n**	**(%)**			**n**	**(%)**			**n**	**(%)**		
Normal	5,184	(64.5)			4,481	(59.4)			4,321	(59.8)		
Borderline	1,022	(12.7)			1,041	(13.8)			1,005	(13.9)		
High	572	(7.1)			573	(7.6)			544	(7.5)		
Very high	1,264	(15.7)			1,447	(19.2)			1,355	(18.8)		
	**Mean**	**(SD)**			**Mean**	**(SD)**			**Mean**	**(SD)**		
CES-D score	15.6	(11.1)	18	(0.2)	16.6	(11.7)	27	(0.4)	16.9	(11.9)	25	(0.3)
	**n**	**(%)**			**n**	**(%)**			**n**	**(%)**		
Low	4,735	(58.8)			4,163	(55.1)			3,932	(54.3)		
At risk	2,100	(26.1)			1,989	(26.3)			1,891	(26.1)		
Caseness	1,219	(15.1)			1,409	(18.6)			1,415	(19.5)		
	**Mean**	**(SD)**			**Mean**	**(SD)**			**Mean**	**(SD)**		
Suicidality	0.4	(0.9)	22	(0.3)	0.5	(1.0)	34	(0.4)	0.5	(1.0)	30	(0.4)
	**n**	**(%)**			**n**	**(%)**			**n**	**(%)**		
Low risk	6,439	(80.0)			5,793	(76.7)			5,457	(75.4)		
Life not worth living	579	(7.2)			569	(7.5)			539	(7.5)		
Self-harm thoughts	433	(5.4)			466	(6.2)			463	(6.4)		
Self-harm behaviors	599	(7.4)			726	(9.6)			774	(10.7)		
	**Mean**	**(SD)**			**Mean**	**(SD)**			**Mean**	**(SD)**		
BRIEF-2 at T1	83.7	(20.8)	1,433	(17.8)	83.4	(20.5)	1,441	(18.9)	83.1	(20.4)	1,365	(18.8)
Behavioral self-regulation	20.7	(5.6)	1,412	(17.5)	20.6	(5.6)	1,422	(18.7)	20.5	(5.5)	1,347	(18.5)
Emotional self-regulation	22.4	(6.2)	1,424	(17.6)	22.3	(6.1)	1,434	(18.9)	22.2	(6.1)	1,359	(18.7)
Cognitive self-regulation	40.6	(10.5)	1,419	(17.6)	40.5	(10.4)	1,427	(18.8)	40.4	(10.3)	1,351	(18.6)
Inhibition	12.9	(3.6)	1,412	(17.5)	12.9	(3.5)	1,422	(18.7)	12.8	(3.5)	1,347	(18.5)
Self-monitoring	7.7	(2.4)	1,231	(15.3)	7.7	(2.4)	1,259	(16.6)	7.7	(2.4)	1,186	(16.3)
Shifting	12.5	(3.6)	1,422	(17.6)	12.5	(3.5)	1,432	(18.9)	12.5	(3.5)	1,357	(18.7)
Emotional control	9.8	(3.2)	1,297	(16.1)	9.8	(3.1)	1,315	(17.3)	9.8	(3.1)	1,241	(17.1)
Task completion	11.3	(3.3)	1,391	(17.2)	11.3	(3.3)	1,403	(18.5)	11.3	(3.3)	1,330	(18.3)
Working memory	13.3	(3.7)	1,372	(17.0)	13.2	(3.6)	1,384	(18.2)	13.2	(3.6)	1,313	(18.1)
Planning	16.0	(4.3)	1,411	(17.5)	16.0	(4.2)	1,420	(18.7)	15.9	(4.2)	1,345	(18.5)

Note: Higher scores on the BRIEF-2 mean greater difficulties. The different possible score ranges for the BRIEF-2 subscales and skills are executive function, 52-156; behavioral self-regulation, 13-39; emotional self-regulation, 14-42; cognitive self-regulation, 25-75; inhibition, 8-24; self-monitoring, 5-15; shifting, 8-24; emotional control, 6-18; task completion, 7-21; working memory, 8-24; and planning, 10-30. The possible score range for suicidality is 0-3. References are in Supplement 1, available online. BRIEF-2 = Behavior Rating Inventory of Executive Function, Second Edition; CES-D = Center for Epidemiological Studies Depression Scale (range 0-60); SDQ = Strengths and Difficulties Questionnaire (range 0-40); WEMWBS = Warwick-Edinburgh Mental Well-Being Scale (range 14-70).

The percentage of missing data for each outcome was low at each time point (<1%) (Table 1). Overall, 7,263 adolescents (90%; 84 schools) remained until T3, and 7,076 (88%) completed questionnaires for at least 1 mental health outcome. Adolescents without (vs with) follow-up data identified more often as male (45% vs 43%) and reported lower T1 levels of mental well-being (mean [SD] = 47.4 [9.8] vs 49.3 [8.9]); higher social-emotional-behavioral difficulties (14.3 [6.8] vs 12.1 [6.6]), depression (17.5 [11.6] vs 15.3 [11.0]), and suicidality (0.6 [1.0] vs 0.4 [0.9]); and poorer executive function (87.9 [22.9] vs 83.1 [20.4]) (Table S2, available online).

Overall, adolescents’ mental health declined over time, particularly among girls (Figure S1, available online). Mean scores for mental well-being, social-emotional-behavioral difficulties, and suicidality were within the normal range. Mean scores for depression were in the range of at risk for depression at all time points. Self-reported executive function was moderately to strongly associated with all outcomes at T1 (Table S3, available online). Exploration of the items underlying the emotional self-regulation dimension and their relationship with mental health outcomes at T1 suggests that although these items were associated with our outcomes—particularly social-emotional-behavioral difficulties and depressive symptoms—they reflect distinct constructs (Table S4, available online). Interaction plots for the univariable effects are shown in Figure S2, available online, and the results of the univariable analyses are shown in Tables S5 through S7, available online.

### Model 1

In multivariable analyses (Table 2), better self-reported executive function at T1 (ie, lower BRIEF-2 scores) was significantly associated with better mental well-being (regression coefficient [*B*] = −.23; 95% CI −0.24, −0.22; *p* < .001), fewer social-emotional-behavioral difficulties (*B* = .24; 95% CI 0.23, 0.24; *p* < .001), and less depression (*B* = .33; 95% CI 0.32, 0.34; *p* < .001) and suicidality (*B* = .02; 95% CI 0.01, 0.02; *p* < .001). These associations were stable over time for suicidality, but weakened for mental well-being, social-emotional-behavioral difficulties, and depression. In other words, although better self-reported executive function at T1 was associated with similar levels of suicidality over time, the association between self-reported executive function and adolescents’ mental well-being, social-emotional-behavioral difficulties, and depression was stronger at T1 compared with subsequent assessments. Over time, levels of mental well-being, social-emotional-behavioral difficulties, and depression were more similar for adolescents with different levels of self-reported executive function at T1. This convergence was driven by decreasing mental well-being and increasing social-emotional-behavioral difficulties and depression in adolescents with higher levels of self-reported executive function at T1 (see Figure S2, available online). These associations were found in girls and boys, but associations with depression and suicidality were stronger in girls (depression: *B* = .37; 95% CI 0.35, 0.38 vs *B* = .25; 95% CI 0.24, 0.27; suicidality: *B* = .02; 95% CI 0.02, 0.02 vs *B* = .01; 95% CI 0.01, 0.01) (Tables S8 and S9, available online).

**Table 2:** Multivariable Analyses of Executive Function at T1 and Mental Health Outcomes (Across T1 to T3) Based on 3-Level Random Intercept Model

	Well-being (range 14-70)			Social-emotional-behavioral difficulties (range 0-40)			Depression (range 0-60)			Suicidality (range 0-3)		
	Coeff.	95% CI	*p*	Coeff.	95% CI	*p*	Coeff.	95% CI	*p*	Coeff.	95% CI	*p*
**Model 1: executive function (total executive function)**												
Executive function	−0.23	−0.24, −0.22	<.001∗	0.24	0.23, 0.24	<.001∗	0.33	0.32, 0.34	<.001∗	0.02	0.01, 0.02	<.001∗
T2 × executive function	0.02	0.01, 0.03	<.001∗	−0.02	−0.03, −0.02	<.001∗	−0.04	−0.05, −0.02	<.001∗	NA	NA	NA
T3 × executive function	0.03	0.02, 0.04	<.001∗	−0.04	−0.04, −0.03	<.001∗	−0.05	−0.06, −0.03	<.001∗	NA	NA	NA
**Model 2: self-regulation**												
Behavioral	0.02	−0.04, 0.09	.433^a^	0.26	0.22, 0.30	<.001∗	−0.04	−0.12, 0.03	.222	0.01	0.00, 0.02	<.001∗
T2 × behavioral	0.05	−0.01, 0.12	.104^a^	−0.04	−0.08, 0.00	.027∗	−0.02	−0.09, 0.05	.545	NA	NA	NA
T3 × behavioral	0.07	0.00, 0.13	.046^a^	−0.06	−0.10, −0.02	.003∗	−0.04	−0.11, 0.03	.261	NA	NA	NA
Emotional	−0.61	−0.67, −0.55	<.001∗^,^^a^	0.47	0.44, 0.51	<.001∗	1.12	1.05, 1.19	<.001∗	0.04	0.04, 0.05	<.001∗
T2 × emotional	0.09	0.03, 0.15	.002∗^,^^a^	−0.11	−0.14, −0.07	<.001∗	−0.24	−0.30, −0.17	<.001∗	NA	NA	NA
T3 × emotional	0.09	0.03, 0.15	.003∗^,^^a^	−0.13	−0.17, −0.10	<.001∗	−0.25	−0.32, −0.18	<.001∗	NA	NA	NA
Cognitive	−0.15	−0.19, −0.11	<.001∗^,^^a^	0.10	0.07, 0.12	<.001∗	0.08	0.04, 0.12	<.001∗	0.00	0.00, 0.01	.029∗
T2 × cognitive	−0.03	−0.07, 0.01	.171^a^	0.03	0.01, 0.06	.006∗	0.07	0.03, 0.11	.002∗	NA	NA	NA
T3 × cognitive	−0.02	−0.06, 0.02	.351^a^	0.02	0.00, 0.05	.043	0.07	0.02, 0.11	.003∗	NA	NA	NA
**Model 3: executive function skills**												
Inhibition	0.17	0.07, 0.26	.001∗	0.37	0.31, 0.43	<.001∗	−0.19	−0.30, −0.07	.001∗	0.00	−0.01, 0.01	.631
T2 × inhibition	0.02	−0.08, 0.12	.729	−0.04	−0.10, 0.02	.149	0.00	−0.11, 0.12	.898	−0.00	−0.01, 0.01	.785
T3 × inhibition	0.02	−0.08, 0.12	.703	−0.06	−0.12, 0.00	.046	0.03	−0.08, 0.15	.574	0.00	−0.01, 0.01	.629
Self-monitoring	−0.16	−0.30, −0.02	.024	0.04	−0.04, 0.12	.349	0.08	−0.07, 0.24	.289	0.02	0.00, 0.03	.010∗
T2 × monitoring	0.11	−0.03, 0.26	.110	−0.04	−0.12, 0.05	.387	−0.06	−0.22, 0.10	.433	NA	NA	NA
T3 × monitoring	0.15	0.01, 0.29	.041	−0.04	−0.13, 0.04	.333	−0.15	−0.31, 0.01	.074	NA	NA	NA
Shifting	−0.32	−0.43, −0.21	<.001∗	0.16	0.09, 0.22	<.001∗	0.37	0.25, 0.49	<.001∗	−0.01	−0.02, 0.00	.103
T2 × shifting	0.12	0.01, 0.22	.040	−0.04	−0.11, 0.02	.210	−0.16	−0.28, −0.03	.013∗	NA	NA	NA
T3 × shifting	0.06	−0.05, 0.17	.272	−0.07	−0.14, 0.00	.037	−0.17	−0.29, −0.04	.009∗	NA	NA	NA
Emotional control	−0.84	−0.93, −0.75	<.001∗	0.74	0.68, 0.79	<.001∗	1.71	1.61, 1.81	<.001∗	0.08	0.07, 0.09	<.001∗
T2 × emotional control	0.08	−0.02, 0.17	.117	−0.16	−0.22, −0.11	<.001∗	−0.30	−0.40, −0.19	<.001∗	NA	NA	NA
T3 × emotional control	0.11	0.02, 0.21	.021	−0.18	−0.24, −0.13	<.001∗	−0.31	−0.42, −0.20	<.001∗	NA	NA	NA
Task completion	−0.07	−0.18, 0.05	.255	0.04	−0.03, 0.10	.277	−0.05	−0.18, 0.07	.410	−0.00	−0.01, 0.01	.519
T2 × task	−0.00	−0.12, 0.11	.974	0.00	−0.07, 0.07	.955	0.04	−0.09, 0.17	.523	NA	NA	NA
T3 × task	0.06	−0.05, 0.18	.292	0.04	−0.03, 0.11	.239	0.10	−0.03, 0.23	.128	NA	NA	NA
Working memory	−0.21	−0.31, −0.11	<.001∗	0.21	0.15, 0.27	<.001∗	0.18	0.07, 0.30	.002∗	0.00	−0.01, 0.01	.454
T2 × working memory	−0.01	−0.12, 0.09	.795	0.02	−0.04, 0.08	.522	0.11	−0.01, 0.22	.069	0.00	0.00, 0.02	.134
T3 × working memory	−0.04	−0.14, 0.07	.480	0.00	−0.06, 0.07	.893	0.15	0.04, 0.27	.011∗	0.00	0.00, 0.02	.072
Planning	−0.28	−0.37, −0.18	<.001∗	0.16	0.10, 0.22	<.001∗	0.37	0.26, 0.48	<.001∗	0.02	0.02, 0.03	<.001∗
T2 × planning	−0.07	−0.17, 0.03	.174	0.04	−0.01, 0.10	.135	0.03	−0.08, 0.14	.608	NA	NA	NA
T3 × planning	−0.06	−0.16, 0.04	.245	0.00	−0.06, 0.06	.905	−0.07	−0.18, 0.04	.220	NA	NA	NA

Note: The unstandardized regression coefficients are presented to show the actual change in the outcome measure associated with a 1-unit change in the predictor. Executive function subdimensions and skills were cluster-mean centered and treated as time-constant (ie, analyzed only at T1). Higher executive function scores (including self-regulation and skills) mean greater difficulties. Models were adjusted for cohort, allocation, age (cluster centered), and ethnicity. Students are nested within schools. T1 is the reference.

Coeff. = coefficient; NA = only the main effect of time, but not the time × predictor interaction term, was included.

∗Significant (*p* < .05) after adjustment for multiple comparisons.

a To address convergence problems, this model was estimated with the BOBYQA optimizer for a quadratic approximation and full maximum likelihood estimation.

### Model 2

Of all 3 self-regulation dimensions, self-reported emotional self-regulation showed the strongest associations with mental well-being (*B* = −.61; 95% CI −0.67, −0.55; *p* < .001), social-emotional-behavioral difficulties (*B* = .47; 95% CI 0.44, 0.51; *p* < .001), depression (*B* = 1.12; 95% CI 1.05, 1.19; *p* < .001), and suicidality (*B* = .04; 95% CI 0.04, 0.05; *p* < .001). That is, better self-reported emotional self-regulation at T1 (ie, lower BRIEF-2 emotional self-regulation scores) was associated with better mental well-being and fewer social-emotional-behavioral difficulties, less depression, and less suicidality. These associations were stable over time for suicidality, but weakened for mental well-being, social-emotional-behavioral difficulties, and depression. In other words, although better self-reported emotional self-regulation at T1 was associated with similar levels of suicidality over time, the association between self-reported emotional self-regulation and mental well-being, social-emotional-behavioral difficulties, and depression was stronger at T1 compared with subsequent assessments. Over time, levels of mental well-being, social-emotional-behavioral difficulties, and depression were more similar for adolescents with different levels of self-reported emotional self-regulation at T1. These associations were found in girls and boys, with stronger associations in girls for mental well-being (*B* = −.59; 95% CI −0.66, −0.51 vs *B* = −.41; 95% CI −0.50, −0.31), depression (*B* = 1.10; 95% CI 1.00, 1.19 vs *B* = .82; 95% CI 0.72, 0.93), and suicidality (*B* = .04; 95% CI 0.04, 0.05 vs *B* = .03; 95% CI 0.02, 0.03) (Tables S8 and S9, available online).

Other significant associations were revealed for self-reported cognitive self-regulation and mental well-being (*B* = −.15; 95% CI −0.19, −0.11; *p* < .001), social-emotional-behavioral difficulties (*B* = .10; 95% CI 0.07, 0.12; *p* < .001), depression (*B* = .08; 95% CI 0.04, 0.12; *p* < .001), and suicidality (*B* < .01; 95% CI 0.00, 0.01; *p* = .029). That is, better self-reported cognitive self-regulation at T1 (ie, lower BRIEF-2 cognitive self-regulation scores) was associated with better mental well-being, fewer social-emotional-behavioral difficulties, less depression, and less suicidality. These associations were stable over time for mental well-being and suicidality, but increased for social-emotional-behavioral difficulties and depression. In other words, better self-reported cognitive self-regulation at T1 was associated with similar levels of mental well-being and suicidality over time, as well as with increasingly fewer social-emotional-behavioral difficulties and less depression over time, after adjustment for cohort, trial arm allocation, age, and ethnicity (Table 2). Similar associations between self-reported cognitive self-regulation and mental well-being, social-emotional-behavioral difficulties, and depression were observed in girls and boys (Tables S8 and S9, available online). However, associations between cognitive self-regulation and suicidality were not found in the subsamples of girls and boys.

Significant associations were found for self-reported behavioral self-regulation and social-emotional-behavioral difficulties (*B* = .26; 95% CI 0.22, 0.30; *p* < .001) and suicidality (*B* = .01; 95% CI 0.00, 0.02; p < .001). That is, better self-reported behavioral self-regulation at T1 (ie, lower BRIEF-2 behavioral self-regulation scores) was associated with fewer social-emotional-behavioral difficulties and less suicidality. These associations weakened over time for social-emotional-behavioral difficulties but were stable for suicidality. In other words, although better self-reported behavioral self-regulation at T1 was associated with similar levels of suicidality over time, the association between self-reported behavioral self-regulation and social-emotional-behavioral difficulties was stronger at T1 compared with subsequent assessments. Over time, levels of social-emotional-behavioral difficulties were more similar for adolescents with different levels of self-reported behavioral self-regulation at T1. Although behavioral self-regulation was associated with social-emotional-behavioral difficulties in girls and boys, the association between behavioral self-regulation and suicidality was evident only in girls (Tables S8 and S9, available online).

### Model 3

**Figure 3:**
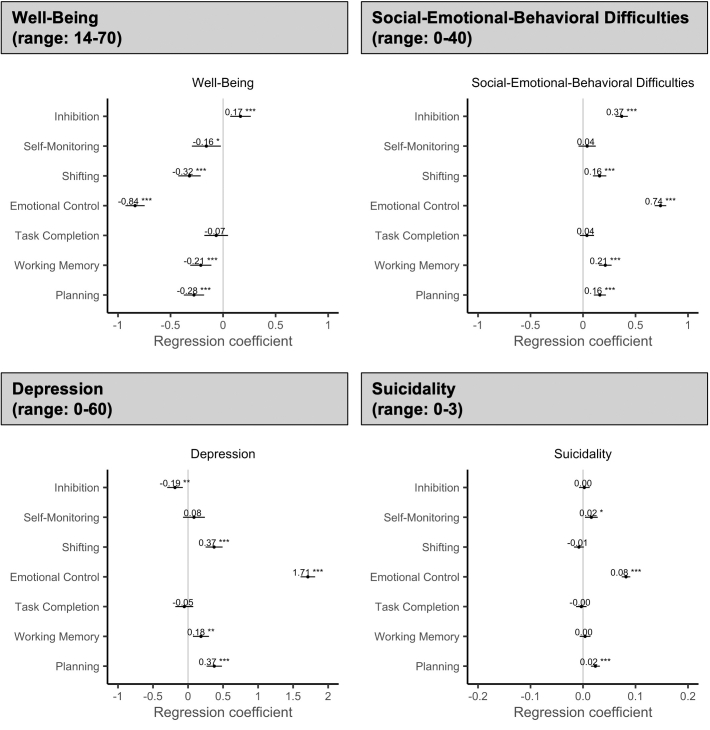
Adjusted Regression Coefficients for the Relation Between Self-Reported Executive Function Skills at T1 and Mental Health Outcomes (T1 to T3) ***Note:****Given the potential problems associated with the standardization of effects in multilevel models, we present the unstandardized regression coefficients. The unstandardized regression coefficients represent the actual change in the outcome measure associated with a 1-unit change in the respective executive function skill, while controlling for all other executive function skills, cohort, allocation, age (cluster centered), and ethnicity (see also*Table 2*). Due to the different possible score ranges of executive function skills and mental health outcomes, these coefficients are not directly comparable and should therefore be interpreted based only on the width of the 95% CI and whether it crosses zero, as an indication of statistical significance and robustness of the reported effects. Higher executive function scores mean greater difficulties.* *∗*p *< .05; ∗∗*p *< .01; ∗∗∗*p *< .001.*

Among the 7 self-reported executive function skills (Figure 3), emotional control was strongly associated with mental well-being (*B* = −.84; 95% CI −0.93, −0.75; *p* < .001), social-emotional-behavioral difficulties (*B* = .74; 95% CI 0.68, 0.79; *p* < .001), depression (*B* = 1.71; 95% CI 1.61, 1.81; *p* < .001), and suicidality (*B* = .08; 95% CI 0.07, 0.09; *p* < .001). That is, better self-reported emotional control at T1 (ie, lower BRIEF-2 emotional control scores) was associated with better mental well-being, fewer social-emotional-behavioral difficulties, less depression, and less suicidality. These associations were stable for mental well-being and suicidality, but weakened over time for social-emotional-behavioral difficulties and depression. In other words, better self-reported emotional control at T1 was associated with similar levels of mental well-being and suicidality over time, but it was more strongly associated with fewer social-emotional-behavioral difficulties and less depression at T1 compared with subsequent assessments. Over time, levels of social-emotional-behavioral difficulties and depression were more similar for adolescents with different levels of self-reported emotional control at T1. These associations were found in girls and boys, but associations with mental well-being and depression were stronger in girls (mental well-being: *B* = −.82; 95% CI −0.94, −0.70 vs *B* = −.51; 95% CI −0.68, −0.34; depression: *B* = 1.67; 95% CI 1.53, 1.81 vs *B* = 1.30; 95% CI 1.13, 1.47) (Tables S8-S10, available online).

Significant, stable relationships were found for self-reported planning and mental well-being (*B* = −.28; 95% CI −0.37, −0.18; *p* < .001), social-emotional-behavioral difficulties (*B* = .16; 95% CI 0.10, 0.22; *p* < .001), depression (*B* = .37; 95% CI 0.26, 0.48; *p* < .001), and suicidality (*B* = .02; 95% CI 0.02, 0.03; *p* < .001). That is, better self-reported planning at T1 (ie, lower BRIEF-2 planning scores) was associated with better mental well-being, fewer social-emotional-behavioral difficulties, less depression, and less suicidality over time. These relationships were similar in girls and boys (Tables S8-S10, available online).

Self-reported working memory and shifting were significantly associated with mental well-being (working memory: *B* = −.21; 95% CI −0.31, −0.11; *p* < .001; shifting: *B* = −.32; 95% CI −0.43, −0.21; *p* < .001), social-emotional-behavioral difficulties (working memory: *B* = .21; 95% CI 0.15, 0.27; *p* < .001; shifting: *B* = .16; 95% CI 0.09, 0.22; *p* < .001), and depression (working memory: *B* = .18; 95% CI 0.07, 0.30; *p* = .002; shifting: *B* = .37; 95% CI 0.25, 0.49; *p* < .001). That is, better self-reported working memory and shifting at T1 (ie, lower BRIEF-2 working memory and shifting scores) were associated with better mental well-being, fewer social-emotional-behavioral difficulties, and less depression. These associations were stable over time except for shifting and depression, where the relation weakened. In other words, self-reported shifting was more strongly associated with depression at T1 compared with subsequent assessments. Over time the depression trajectories of adolescents with different levels of shifting skills at T1 converged. For shifting, similar associations were found for girls and boys (Tables S8-S10, available online). For working memory, girls reported significant associations with mental well-being, social-emotional-behavioral difficulties, and depression, whereas boys reported significant associations only with social-emotional-behavioral difficulties (Tables S8-S10).

Self-reported inhibition was significantly associated with mental well-being (*B* = .17; 95% CI 0.07, 0.26; *p* < .001), social-emotional-behavioral difficulties (*B* = .37; 95% CI 0.31, 0.43; *p* < .001), and depression (*B* = −.19; 95% CI −0.30, −0.07; *p* < .001), which were stable relationships. That is, better self-reported inhibition at T1 (ie, lower BRIEF-2 inhibition scores) was associated with worse mental well-being, fewer social-emotional-behavioral difficulties, and higher levels of depression over time. In girls and boys, these associations were found only for social-emotional-behavioral difficulties (Tables S8-S10, available online).

Self-reported self-monitoring was associated with suicidality (*B* = .02; 95% CI 0.00, 0.03; *p* = .010), which was a stable relationship. That is, better self-reported self-monitoring at T1 (ie, lower BRIEF-2 self-monitoring scores) was associated with less suicidality. This association was found only in girls. Additionally, for girls, an association between self-monitoring and mental well-being was revealed (Tables S8-S10, available online). Self-reported task completion was not associated with the measured mental health outcomes in adolescence.

## Discussion

Psychological theories highlight the crucial role of executive function in mental health and well-being, suggesting the potential mental health benefit of improving executive function skills through training.^10^^,^^37^^,^^38^ As executive function encompasses a broad set of skills, and most prior studies have focused either on the overall construct of executive function or on specific skills in isolation, the relative importance of specific executive function skills in relation to specific mental health outcomes, ranging from well-being to suicidality, remains poorly understood.^14^^,^^17^ Consequently, it remains unclear which executive function skills such training should address to optimize intervention, prevention, and mental health promotion during the critical years of early/middle adolescence.^5^ This study explored the longitudinal relationships between self-reported executive function (underpinning subdimensions and skills) and adolescents’ mental well-being, social-emotional-behavioral difficulties, depression, and suicidality over 1 year along with potential gender differences. We found that self-reported executive function, especially better emotional self-regulation and emotional control, was associated with better mental health over time. Findings support previous research showing a prospective relationship between higher emotional reactivity (ie, less emotional control) in early childhood and subsequent internalizing difficulties, even after adjusting for important risk factors such as family adversity and peer problems.^17^ Associations were stronger in girls for most outcomes, potentially due to increasing gender disparities in mental health outcomes during adolescence, with alarming rates of depression and self-harm in adolescent girls in recent years.^6^, ^7^, ^8^^,^^39^ One explanation for the greater self-harm rates in girls was their greater levels of psychological distress.^40^ Our findings build on research showing that the (in)ability to tolerate and cope effectively with negative emotions might be as important as the negative emotion itself in driving suicidality.^41^ Findings are in keeping with previous research^20^^,^^21^ suggesting that training to respond effectively to negative emotions by enhancing emotional self-regulation and control might improve mental health outcomes in adolescence, from well-being to suicidality.

Additionally, self-reported cognitive self-regulation, specifically planning, was associated with better mental well-being, fewer social-emotional-behavioral difficulties, less depression, and less suicidality. Associations for planning were stable over time, with no gender differences. This finding is consistent with previous research that revealed an association between planning difficulties in childhood and an increasing probability of internalizing difficulties in early adolescence.^17^ Planning involves breaking down complex tasks, setting priorities, and anticipating obstacles.^10^ Safety planning is a common and crucial technique used for managing suicidal risk in adolescents.^42^ Our findings highlight the potential benefit of enhancing planning skills to support adolescents’ mental health, especially amid recent uncertainties driven by academic disruptions, food insecurity, familial stress, and social isolation.^39^

Early/middle adolescence is a critical time for the onset of mental health difficulties,^1^^,^^2^ particularly in girls.^6^ Recent studies emphasize the importance of considering gender differences in the development of executive functions and associations with mental health outcomes in early adolescence, as girls may be more vulnerable than boys to the negative effects of early life stress on executive function.^34^^,^^43^ Our findings revealed gender differences in the associations between self-reported working memory and some mental health outcomes. In girls, better self-reported working memory was associated with better mental well-being, fewer social-emotional-behavioral difficulties, and less depression. In boys, this association was observed only for social-emotional-behavioral difficulties.

These findings build on previous research, showing gender differences in the development of working memory,^44^ with advantages in verbal working memory in girls and visuospatial working memory in boys.^45^ However, as effects were in a similar direction for boys and girls, and girls reported higher levels of mental health difficulties at T1, the significant findings in girls may reflect a broader range of responses, increasing the likelihood of detecting an effect in girls despite a qualitatively similar pattern in boys. Similarly, self-reported self-monitoring was uniquely associated with mental well-being and suicidality in girls. Self-monitoring requires awareness of the social context to regulate one’s thoughts, emotions, and behaviors to respond adaptively. Previous research identified distinct developmental windows of sensitivity to negative social contexts, particularly social media, with adolescent girls being particularly affected in early adolescence (11-13 years of age).^46^ Future research should consider longer follow-up periods into late adolescence to establish whether similar effects of self-reported working memory and self-monitoring on mental health outcomes may simply emerge later for boys or whether these effects are qualitatively different for boys and girls. Together, these findings emphasize the need to train executive function skills, particularly emotional control and planning, to support the mental health and well-being of adolescent girls and boys.

These relationships are likely complex and bidirectional.^47^ For some, self-reported emotional control may be directly associated with fewer mental health symptoms (low mood, self-harm/suicidal thoughts), and planning skills may directly relate to less suicidality (eg, through the ability to identify appropriate responses in a future crisis). For others, planning skills may indirectly impact mental health outcomes through better school performance. Additionally, their mood may impact their perception of executive function skills (youth who may feel down may also perceive themselves to be less able to control their emotions or plan effectively). Addressing adolescents’ executive function skills (emotional control and planning) and perceptions thereof through interventions may break this cycle, potentially improving mental health and well-being over time.

This study has considerable strengths, including the longitudinal design, robust measures, and focus on modifiable executive function skills and multiple mental health outcomes ranging from well-being to suicidality. Rigorous data collection resulted in minimal missing data, which together with the representative sample increases confidence in the generalizability of our findings. Limitations include the use of binary gender comparisons, given the low proportion of nonbinary youth (2%). Future research focusing on nonbinary youth is warranted, given the higher rates of mental health difficulties and executive function difficulties observed in this group. The study period of 1 year during early/middle adolescence allowed for an exploration of only short-term mental health trajectories. As executive function skills continue to develop during this stage, cross-sectional relationships between executive function and mental health outcomes may change over time. Nonetheless, we used rigorous statistical methods to investigate prospective relationships, aiming to identify targets for mental health promotion strategies. We used complete case analyses given the low rates of missingness, noting that adolescents without follow-up data were often male and reported lower baseline mental health. Consistent with research highlighting the bidirectional relationship between executive function and mental health difficulties,^47^ it is plausible that associations would be even stronger among individuals with higher levels of mental health difficulties. Therefore, finding associations between self-reported executive function at T1 and mental health outcomes over time in this healthier retained sample suggests that findings are likely robust in the full sample. Finally, feasibility constraints meant that we relied on self-reported executive function measures, which might be influenced by participants’ mental health,^47^ but can be administered in population-based surveys. Previous research has highlighted inconsistences between self-report and task-based assessments of executive function in relation to mental health outcomes in adolescence. Some studies suggest that worse executive function measured via performance-based tasks in childhood is associated with future externalizing and internalizing problems.^14^ However, other studies have found associations between executive function and mental health outcomes in adolescence only for self-report and not for task-based measures,^48^ with often only weak correlations between self-report and task-based measures in young people.^49^ These discrepancies are not solely attributable to measurement error.^49^ Instead, they suggest that task-based and self-report measures assess distinct, though complementary, aspects of executive functioning.^49^^,^^50^ Task-based measures may offer greater objectivity but often lack ecological validity, whereas self-report measures may require more metacognitive awareness.^35^ Future research should consider both self-report and task-based measures to better understand the relationship between executive function and mental health outcomes in adolescence.^49^ Although executive function was measured exclusively with the BRIEF-2 self-report questionnaire in our study, a previous review identified this questionnaire as one of the most widely used measures for assessing executive function in adolescents, with strong psychometric properties, including good to excellent reliability and proven construct, concurrent, and discriminant validity.^35^ Our findings show similarly robust psychometric properties, providing support for the use of the BRIEF-2 as a self-report measure in our study.

In conclusion, we found that better self-reported executive function was significantly associated with better mental health in early/middle adolescence. This association weakened over time for all mental health outcomes except suicidality. Our findings highlight emotional control and planning as promising targets for mental health intervention, prevention, and promotion strategies in adolescent boys and girls.

## CRediT authorship contribution statement

**Verena Hinze:** Writing – original draft, Methodology, Conceptualization, Writing – review & editing, Validation, Formal analysis, Visualization, Investigation. **Sarah-Jayne Blakemore:** Writing – review & editing, Funding acquisition, Investigation, Methodology, Conceptualization. **Tim Dalgleish:** Investigation, Methodology, Conceptualization, Writing – review & editing, Funding acquisition. **Tamsin Ford:** Methodology, Conceptualization, Writing – review & editing, Funding acquisition, Investigation. **Karen L. Mansfield:** Writing – review & editing, Validation. **Obioha C. Ukoumunne:** Validation, Investigation, Writing – review & editing, Methodology, Conceptualization, Supervision, Funding acquisition. **Willem Kuyken:** Validation, Methodology, Data curation, Writing – review & editing, Project administration, Funding acquisition, Supervision, Investigation, Conceptualization. **Jesus Montero-Marin:** Validation, Investigation, Writing – original draft, Methodology, Writing – review & editing, Supervision, Conceptualization.
