# Supplementary material for: The Longitudinal Relationship Between Self-Reported Executive Function and Mental Health in Early Adolescence
Source: JAACAP Open. 2025 Aug 6;4(1):53–67. doi: 10.1016/j.jaacop.2025.07.003 (PMC12925927; doi:10.1016/j.jaacop.2025.07.003)
Supplement: Supplementary file 1 [file mmc1.pdf]

Supplementary Material

The longitudinal relationship between self-reported executive function and mental health in early adolescence

Summary of Supplements

Supplement 1. Additional information on study measures

Supplement 2. Key R packages used for the statistical analyses (along with further dependencies relevant for the performance of these packages)

Table S1. Participant characteristics at T1 by gender identification (N=8072)

Table S2. Participant characteristics at T1 by follow-up status (N=8072)

Figure S1. Adolescents' mental health trajectories over time

Table S3. Pearson correlations for the cross-sectional (T1) relationships between executive function and mental health

Table S4. Spearman correlations for the cross-sectional (T1) relationships between emotional self-regulation and mental health

Figure S2. Interaction plots for the univariable analyses based on the three-level random intercept model

Table S5. Univariable analyses (unadjusted), based on the three-level random intercept model

Table S6. Univariable analyses (unadjusted) for girls, based on the three-level random intercept model

Table S7. Univariable analyses (unadjusted) for boys, based on the three-level random intercept model

Table S8. Multivariable analyses for girls, based on the three-level random intercept model

Table S9. Multivariable analyses for boys, based on the three-level random intercept model

Table S10. Adjusted regression coefficients for the relationship between executive function skills at T1 and mental health outcomes (T1 to T3) by gender, based on the three-level random intercept model

References for the supplements

Supplement 1. Additional information on study measures.

Well-being. The Warwick-Edinburgh Mental Well-Being Scale (WEMWBS; Tennant et al., 2007) was used to measure mental well-being in the past two weeks. Each of the 14 items was scored on a scale from 1 ("none of the time") to 5 ("all of the time"), leading a total score between 14 to 70, with higher scores reflecting greater mental well-being. We treated mental well-being as a continuous variable and used the following cut-off values to aid interpretation: probable mental health difficulties (0-40), possible mental health difficulties (41-44), average well-being (45-59), and high well-being (60-70) (Warwick Medical School, 2021). The WEMWBS is a valid and reliable measure of well-being in adolescents (Clarke et al., 2011). The internal consistency (Cronbach's alpha) of the WEMWBS in our study was $\alpha = 0.87$ at T1, $\alpha = 0.89$ at T2, and $\alpha = 0.91$ at T3.

Social-emotional-behavioral difficulties. Social-emotional-behavioral difficulties in the past six months were assessed with the Strength and Difficulties Questionnaire (SDQ; Goodman, 2001). The SDQ consists of 25 items, which are scored on a scale from 0 ("not true") to 2 ("certainly true") and can be grouped into five subscales to measure emotional symptoms, conduct problems, hyperactivity/inattention, peer problems, and prosocial behavior. The first four subscales can be combined into a total scale (0-40), with a higher score reflecting more difficulties. We treated social-emotional-behavioral difficulties as a continuous variable and used the following cut-off values to aid interpretation: normal (0–14), borderline (15–17), high (18–19), and very high (20–40) (Youth in Mind, 2016). The SDQ is a valid and reliable instrument to measure social-emotional-behavioral difficulties in adolescents (Goodman, 2001). The internal consistency of the SDQ in our study was $\alpha = 0.84$ at T1, $\alpha = 0.85$ at T2, and $\alpha = 0.85$ at T3.

Depression. Depressive symptoms in the past week were assessed with the Center for Epidemiologic Studies-Depression Scale (CESD; Radloff, 1977; Radloff, 1991). The CESD Scale consists of 20 symptom items, which are scored on a scale from 0 ("rarely or none of the time") to 3 ("most or all of the time"), leading to a total score between 0 to 60. Higher total scores reflect greater depressive symptom severity. We treated depression as a continuous variable and used the following cut-off values to aid interpretation: normal (score: 0-15), at risk (score: 16-27) and caseness (score: 28-60; Radloff, 1991). The CESD has been validated and is a reliable screening instrument often used in epidemiological research with adolescents (Dierker et al., 2001; Garrison, Addy, Jackson, McKeown, & Waller, 1991; Roberts, Andrews, Lewinsohn, & Hops, 1990). The internal consistency of the CESD in our study was $\alpha = 0.91$ at T1, $\alpha = 0.92$ at T2, and $\alpha = 0.92$ at T3.

Suicidality. Suicidality was measured, using three standardized questions: "Have you thought that life was not worth living, or that you would be better off dead?", "Have you thought seriously about trying to harm yourself in some way (for example by cutting yourself or taking an overdose of pills or other medication)?", and "Have you actually, deliberately harmed yourself in some way (for example by cutting yourself or taking an overdose of pills or other medication)?". Questions referred to the period since the last assessment (six months to one year). Adolescents responded with 'yes', 'no' or 'prefer not to say'. The response option 'prefer not to say' was conservatively coded as 'no'. These questions were developed specifically for this study (Kuyken et al., 2017) by adapting similar questions previously used in the Avon Longitudinal Study of Parents and Children [ALSPAC] birth cohort in England (Kidger et al., 2012). We combined these three dichotomous variables into one suicidality variable to reflect increasing levels of risk based on the most severe form of suicidal risk, reported by the student. Consistent with previous research (Kirtley, Hussey, & Marzano, 2021), we conceptualized suicidality as a spectrum of increasing severity (0=control (low risk), 1=life was not worth living, 2=self-harm thoughts, 3=self-harm behaviors) and therefore aimed to include it as a continuous variable in our analyses. We described the distribution of this variable (see below: self-harm in the MYRIAD trial across time). To establish whether these three dichotomous variables reflect an underlying continuous latent variable, we created an ordinal variable to reflect our hypothesized levels of increasing severity (0=control (low risk), 1=life was not worth living, 2=self-harm thoughts, 3=self-harm behaviors). As an intuitive effect size metric, we calculated the Guttman Error Rate (G), which is the proportion of adolescents who reported violations of this order (e.g., adolescents who reported self-harm behaviors but no self-harm thoughts). Additionally, we calculated the normalized Guttman Error Rate ($G^* = G$ / number of items) to allow for comparability between studies. As a measure of robustness, we computed bootstrapped (N=2000) 95% confidence intervals via case removal. Results showed that only a very small proportion of adolescents violated our assumption that suicidality can be treated as a continuous variable of increasing severity (T1: G=0.044, 95% CI=[0.039, 0.048]; G*=0.015, 95% CI=[0.013, 0.016]; T2: G=0.040, 95% CI=[0.036, 0.044]; G*=0.013, 95% CI=[0.012, 0.015]; T3: G=0.039, 95% CI=[0.035, 0.043]; G*=0.013, 95% CI=[0.012, 0.014]). This finding is consistent with previous research (Kirtley et al., 2021) and supports our approach of treating suicidality, as a continuous variable of increasing severity (0-3). The internal consistency of this scale was $\alpha = 0.76$ at T1, $\alpha = 0.80$ at T2, and $\alpha = 0.81$ at T3.

Self Harm in the MYRIAD trial across time

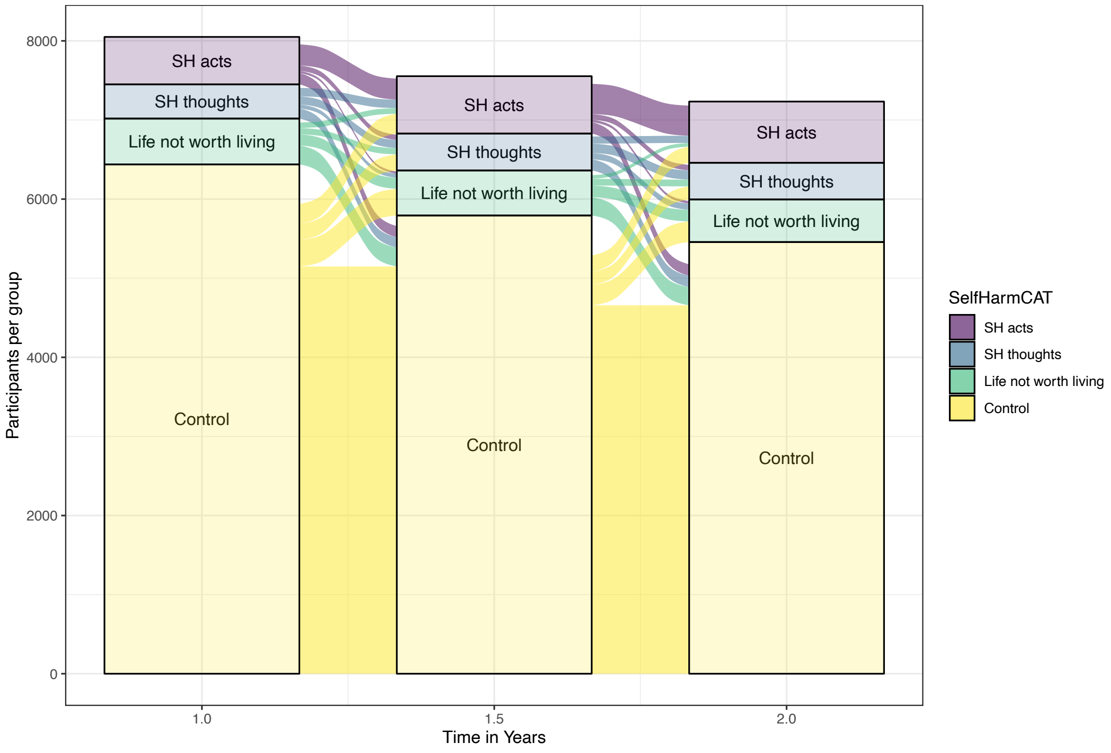

T1 (K=84; N=8072)

group	n
Controls	6439
Life not worth living	579
SH thoughts	433
SH acts	599
NA	326

T2 (K=84; N=7588)

group	n
Controls	5793
Life not worth living	569
SH thoughts	466
SH acts	726
NA	822

T3 (K=84; N=7263)

group	n
Controls	5457
Life not worth living	539
SH thoughts	463
SH acts	774
NA	1143

T1 (K=84; N=8072)

Guttman errors

T1_Self_01b	T1_Self_02b	T1_Self_03b	n	guttman_error
No	No	No	6437	FALSE
Yes	No	No	579	FALSE
Yes	Yes	Yes	377	FALSE
NA	NA	NA	326	NA
Yes	Yes	No	288	FALSE
No	Yes	No	144	TRUE
No	No	Yes	98	TRUE
No	Yes	Yes	88	TRUE
Yes	No	Yes	36	TRUE
No	NA	NA	1	NA
NA	No	No	1	NA
NA	Yes	No	1	NA

Error rate and bootstrapped 95%CIs

metric	median	ci_lwr	ci_upr
guttman_error_rate	0.044	0.039	0.048
normalized_guttman_error_rate	0.015	0.013	0.016

T2 (K=84; N=7588)

Guttman errors

T2_Self_01b	T2_Self_02b	T2_Self_03b	n	guttman_error
No	No	No	5792	FALSE
NA	NA	NA	822	NA
Yes	No	No	568	FALSE
Yes	Yes	Yes	516	FALSE
Yes	Yes	No	342	FALSE
No	Yes	No	124	TRUE
No	No	Yes	93	TRUE
No	Yes	Yes	81	TRUE
Yes	No	Yes	36	TRUE
Yes	NA	NA	1	NA
NA	No	No	1	NA

Error rate and bootstrapped 95%CIs

metric	median	ci_lwr	ci_upr
guttman_error_rate	0.040	0.036	0.044
normalized_guttman_error_rate	0.013	0.012	0.015

T3 (K=84; N=7263)

Guttman errors

T3_Self_01b	T3_Self_02b	T3_Self_03b	n	guttman_error
No	No	No	5456	FALSE
NA	NA	NA	1143	NA
Yes	Yes	Yes	558	FALSE
Yes	No	No	539	FALSE
Yes	Yes	No	356	FALSE
No	Yes	Yes	108	TRUE
No	Yes	No	107	TRUE
No	No	Yes	73	TRUE
Yes	No	Yes	35	TRUE
NA	No	No	1	NA

Error rate and bootstrapped 95%CIs

metric	median	ci_lwr	ci_upr
guttman_error_rate	0.039	0.035	0.043
normalized_guttman_error_rate	0.013	0.012	0.014

SH=self-harm.

Executive function. Executive function in the past six months was measured with the Behavior Rating Inventory of Executive Function, Second Edition, Self-Report Form (BRIEF-2; Gioia, Isquith, Guy, & Kenworthy, 2015). The BRIEF-2 consists of 55 items that are rated on a three-point scale (1=”never”, 2=”sometimes”, 3=”often”). Three items (“I forget my name”, “I have trouble counting to three”, and “I cannot find the front door of my home”) are included as a measure of validity and do not contribute to the calculation of total sum scores, i.e., the composite executive function score (range 52-156). Higher scores reflect greater executive function difficulties. This total score is composed of three self-regulation dimensions, which in turn consist of seven executive skills: behavioral (inhibition & self-monitoring), emotional (shifting & emotional control), and cognitive self-regulation (task completion, working memory, & planning). We replicated the BRIEF-2 factor structure using confirmatory factor analysis and the robust maximum likelihood estimator (Comparative Fit Index [CFI]=0.900, Tucker-Lewis Index [TLI]=0.895, Root Mean Square Error of Approximation [RMSEA] (90% confidence interval)=0.039 (0.039-0.040), Standardized Root Mean Square Residual [SRMR]=0.042). We explored executive function and its subdimensions separately to obtain a more detailed understanding of the relationship between executive function and mental health. All executive function measures were treated as time-constant, continuous variables, measured at T1. For further information on the internal consistencies see below:

Reliability: Internal consistencies of executive function at T1.

	**α [95% confidence interval]**
**Total executive function**	0.97 [0.97; 0.97]
**Self-regulation**	
Behavioral	0.89 [0.89; 0.89]
Emotional	0.91 [0.90; 0.91]
Cognitive	0.94 [0.94; 0.94]
**Executive function skills**	
Inhibition	0.83 [0.83; 0.84]
Self-monitoring	0.79 [0.78; 0.79]
Shifting	0.86 [0.86; 0.87]
Emotional control	0.84 [0.84; 0.85]
Task completion	0.86 [0.86; 0.86]
Working memory	0.84 [0.83; 0.84]
Planning	0.85 [0.85; 0.86]

Validity: Item-Pair Correlations and Absolute Difference Scores at T1.

Item pairs		Expected $r^{\dagger}$	Observed r [95% confidence interval]‡
1. I have trouble sitting still	12. I have a short attention span	0.47	0.47 [0.45; 0.49]***
6. I have angry outbursts	14. I have outbursts for little reason	0.63	0.50 [0.48; 0.52]***
22. I get upset over small events	27. I overreact	0.59	0.46 [0.44; 0.48]***
23. I have good ideas but do not get the job done (I lack follow-through)	52. I have trouble carrying out the things that are needed to reach a goal (such as saving money for special items or studying to get good grades)	0.52	0.41 [0.39; 0.43]***
28. I have trouble remembering things, even for a few minutes (such as directions or phone numbers)	41. I forget instructions easily	0.59	0.53 [0.51; 0.55]***
33. I am slower than others when completing my work	42. It takes me longer to complete my work	0.75	0.63 [0.61; 0.64]***
44. I have problems completing my work	55. I have problems finishing long-term projects (such as papers or book reports)	0.60	0.49 [0.45; 0.49]***
45. I have trouble thinking of a different way to solve a problem when I get stuck	53. I have difficulty coming up with different ways of solving a problem	0.66	0.61 [0.59; 0.63]***

†Provided in the BRIEF-2 manual (Gioia, Isquith, Guy, & Kenworthy, 2015).
‡Two-sided 95% bootstrap confidence interval for the Spearman correlation coefficient based on 10000 bootstrap replications and the bca method. ***p<0.001.

Protocol Classification	Absolute difference scores: N (proportion)
Acceptable (≦5)	6591 (99.3%)
Questionable (7)	44 (0.7%)
Inconsistent (≧8)	4 (0.1%)

Note. This is consistent with the inconsistency score percentiles for typically developing young people (n=803) reported in the BRIEF-2 manual: 97% reported a score of ≦5 (Gioia, Isquith, Guy, & Kenworthy, 2015).

Supplement 2. Key R packages used for the statistical analyses (along with further dependencies relevant for the performance of these packages).

Analysis part	R package (version number)	Reference
Descriptive statistics	dplyr (1.0.8)	Wickham, François, Henry, and Müller (2022)
	Hmisc (4.6-0)	Harrell (2021)
	psych (2.1.9)	Revelle (2021)
	base (3.6.2)	R Core Team (2019)
Data preparation	tidyr (1.2.0)	Wickham & Girlich (2022)
	tidyverse (1.3.1)	Wickham et al., (2019)
	misty (0.4.3)	Yanagida (2021)
	readspss (0.14)	Garbuszus & Pfaff (2021)
Graphical visualizations	ggplot2 (3.3.5)	Wickham (2016)
	sjPlot (2.8.9)	Lüdecke (2021)
	graphics (3.6.2)	R Core Team (2019)
	viridis (0.6.2)	Garnier, Ross, Rudis, Camargo, Sciaini, & Scherer (2021)
	ggalluvial (0.12.3)	Brunson (2020); Brunson & Read (2020)
Multilevel growth analysis	lme4 (1.1-27.1)	Bates, Maechler, Bolker, & Walker (2015)
	car (3.0-12)	Fox, & Weisberg (2019)
	parameters (0.15.0)	Lüdecke, Ben-Shachar, Patil, & Makowski (2020)
Assumptions/ correction for multiple comparisons/ ordinary linear regression analysis/ Correlations	stats (3.6.2)	R Core Team (2019)
	performance (0.8.0)	Lüdecke, Ben-Shachar, Patil, Waggoner, & Makowski, (2021)

Table S1. Participant characteristics at T1 by gender identification (N=8072).

Variables	Female (N = 4380)	Male (N = 3389)	Other* (N = 158)
Age, mean (SD)	13.1 (0.6)	13.2 (0.6)	13.1 (0.6)
Ethnicity – White [a], n (%)	3170 (72.6)	2680 (79.5)	117 (74.5)
Well-being [b], mean (SD)	47.9 (9.2)	50.8 (8.5)	45.4 (10.7)
Social-emotional-behavioral difficulties [c], mean (SD)	13.0 (6.8)	11.5 (6.3)	14.9 (7.2)
Depression [d], mean (SD)	17.6 (11.9)	12.8 (9.2)	19.3 (12.1)
Suicidality [e], mean (SD)	0.5 (1.0)	0.3 (0.8)	0.7 (1.1)
Executive function [f], mean (SD)	85.1 (20.7)	81.4 (20.6)	89.6 (20.8)
Behavioral self-regulation [g], mean (SD)	20.7 (5.6)	20.6 (5.7)	22.3 (6.0)
Emotional self-regulation [h], mean (SD)	23.4 (6.3)	20.9 (5.8)	24.0 (6.0)
Cognitive self-regulation [i], mean (SD)	41.1 (10.4)	39.9 (10.6)	43.3 (10.8)
Inhibition [j], mean (SD)	13.0 (3.6)	12.9 (3.6)	14.0 (3.9)
Self-monitoring [k], mean (SD)	7.7 (2.4)	7.7 (2.4)	8.3 (2.6)
Shifting [l], mean (SD)	12.9 (3.6)	12.1 (3.5)	13.5 (3.4)
Emotional control [m], mean (SD)	10.5 (3.3)	8.8 (2.7)	10.5 (3.2)
Task completion [n], mean (SD)	11.4 (3.3)	11.2 (3.4)	12.0 (3.5)
Working memory [o], mean (SD)	13.5 (3.7)	13.0 (3.7)	14.0 (3.7)
Planning [p], mean (SD)	16.2 (4.3)	15.8 (4.3)	17.2 (4.5)

Note. Please note that higher executive function scores mean greater difficulties. Please also note the different possible score ranges for the executive function subscales and skills: executive function (range: 52-156), behavioral self-regulation (range: 13-39), emotional self-regulation (range: 14-42), cognitive self-regulation (range: 25-75), inhibition (range: 8-24), self-monitoring (range: 5-15), shifting (range: 8-24), emotional control (range: 6-18), task completion (range: 7-21), working memory (range: 8-24), and planning (range: 10-30).
* Defined as those adolescents, responded with 'other' or 'prefer not to say' to the gender question.
[a] Sample size for females: 4369. Sample size for males: 3372. Sample size for other: 157.
[b] Sample size for females: 4372. Sample size for males: 3384. Sample size for other: 157.
[c] Sample size for females: 4372. Sample size for males: 3368.
[b] Sample size for females: 4373. Sample size for males: 3378.
[e] Sample size for females: 4371. Sample size for males: 3376.
[f] Sample size for females: 3709. Sample size for males: 2727. Sample size for other: 137.
[g] Sample size for females: 3721. Sample size for males: 2736. Sample size for other: 137.
[h] Sample size for females: 3711. Sample size for males: 2734. Sample size for other: 137.
[i] Sample size for females: 3718. Sample size for males: 2732. Sample size for other: 137.
[j] Sample size for females: 3721. Sample size for males: 2736. Sample size for other: 137.
[k] Sample size for females: 3817. Sample size for males: 2813. Sample size for other: 138.
[l] Sample size for females: 3712. Sample size for males: 2735. Sample size for other: 137.
[m] Sample size for females: 3784. Sample size for males: 2786. Sample size for other: 137.
[n] Sample size for females: 3730. Sample size for males: 2747. Sample size for other: 138.
[o] Sample size for females: 3740. Sample size for males: 2756. Sample size for other: 137.
[p] Sample size for females: 3723. Sample size for males: 2735. Sample size for other: 137.

Table S2. Participant characteristics at T1 by follow-up status (N=8072).

Variables	Adolescents lost to follow-up* (N = 996)	Remaining adolescents** (N = 7076)
Age, mean (SD)	13.1 (0.6)	13.1 (0.6)
Gender [a]		
Female, n (%)	516 (52.9)	3864 (55.6)
Male, n (%)	434 (44.5)	2955 (42.5)
Other, n (%)	6 (0.6)	19 (0.3)
Prefer not to say, n (%)	20 (2.0)	113 (1.6)
Ethnicity – White [b], n (%)	724 (74.6)	5243 (75.7)
Well-being [c], mean (SD)	47.4 (9.8)	49.3 (8.9)
Social-emotional-behavioral difficulties [d], mean (SD)	14.3 (6.8)	12.1 (6.6)
Depression [e], mean (SD)	17.5 (11.6)	15.3 (11.0)
Suicidality [f], mean (SD)	0.6 (1.0)	0.4 (0.9)
Executive function [g], mean (SD)	87.9 (22.9)	83.1 (20.4)

Note. Please note that higher executive function scores mean greater difficulties.
* Defined as those adolescents with missing data on all 4 outcomes at follow-up (T3).
** Defined as those adolescents with at least one of the outcomes at follow-up (T3).
[a] Sample size in lost to follow-up group: 976. Sample size those remaining: 6951.
[b] Sample size in lost to follow-up group: 971. Sample size those remaining: 6929.
[c] Sample size in lost to follow-up group: 993. Sample size those remaining: 7065.
[d] Sample size in lost to follow-up group: 989. Sample size those remaining: 7053.
[e] Sample size in lost to follow-up group: 991. Sample size those remaining: 7063.
[f] Sample size in lost to follow-up group: 992. Sample size those remaining: 7058.
[g] Sample size in lost to follow-up group: 750. Sample size those remaining: 5889.

Figure S1. Adolescents' mental health trajectories over time.

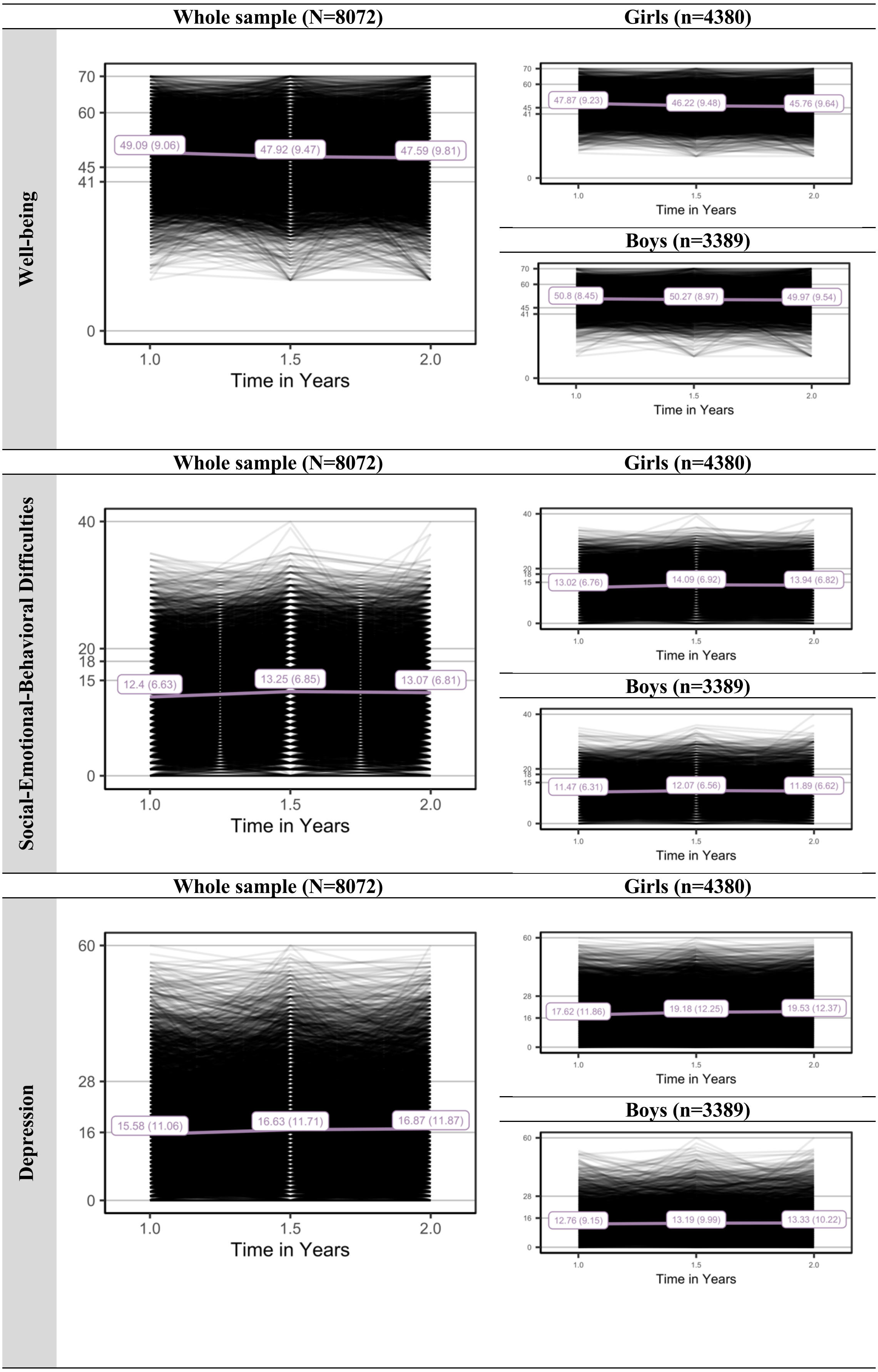

	Whole sample (N=8072)	Girls (n=4380)
**Suicidality**	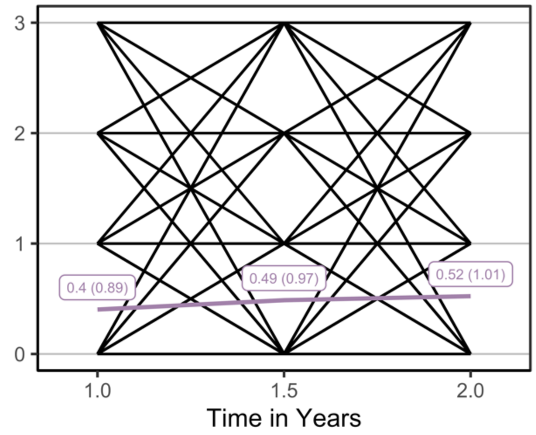	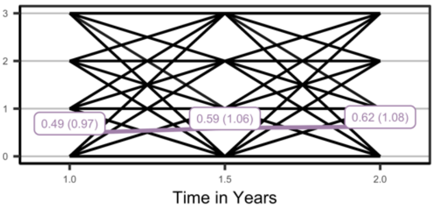
		**Boys (n=3389)**
		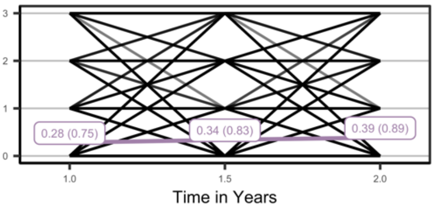

Note. Cut-off scores are based on the official scoring guidelines.
Well-being: probable mental health difficulties (0-40); possible mental health difficulties (41-44); average mental well-being (45-59); high well-being (60-70; Tennant et al., 2007; Warwick Medical School, 2021).
Social-emotional-behavioral difficulties: normal (0–14); borderline (15–17); high (18–19); very high (20–40; Youth in Mind, 2016).
Depression: low (0–15); at risk of depression (16–27); caseness (28–60; Radloff, 1977; Radloff, 1991).
Suicidality: cut-off values: no suicidal risk (0); live not worth living (1); self-harm thoughts (2); self-harm behaviors (3).

Table S3. Pearson correlations for the cross-sectional (T1) relationships between executive function and mental health.

		WEMWBS	SDQ	CESD	Suicidality
**T1**	Sample	r [95%CI]	r [95%CI]	r [95%CI]	r [95%CI]
**Executive function**	*Total*	-0.54 [-0.55, -0.52]	0.74 [0.73, 0.75]	0.62 [0.60, 0.63]	0.35 [0.32, 0.37]
	Girls	-0.58 [-0.60, -0.56]	0.77 [0.75, 0.78]	0.65 [0.63, 0.67]	0.37 [0.34, 0.40]
	Boys	-0.47 [-0.50, -0.44]	0.71 [0.69, 0.73]	0.57 [0.54, 0.59]	0.29 [0.25, 0.32]
**Self-regulation**					
Behavioral	*Total*	-0.44 [-0.46, -0.42]	0.67 [0.66, 0.69]	0.50 [0.49, 0.52]	0.30 [0.28, 0.32]
	Girls	-0.49 [-0.51, -0.46]	0.69 [0.67, 0.71]	0.55 [0.53, 0.57]	0.33 [0.30, 0.36]
	Boys	-0.39 [-0.42, -0.36]	0.66 [0.64, 0.68]	0.48 [0.45, 0.51]	0.24 [0.21, 0.28]
Emotional	*Total*	-0.55 [-0.57, -0.53]	0.73 [0.72, 0.74]	0.67 [0.65, 0.68]	0.36 [0.34, 0.38]
	Girls	-0.58 [-0.60, -0.56]	0.75 [0.73, 0.76]	0.69 [0.67, 0.70]	0.37 [0.35, 0.40]
	Boys	-0.46 [-0.49, -0.43]	0.69 [0.67, 0.71]	0.60 [0.57, 0.62]	0.30 [0.26, 0.33]
Cognitive	*Total*	-0.50 [-0.52, -0.49]	0.69 [0.67, 0.70]	0.56 [0.55, 0.58]	0.31 [0.29, 0.33]
	Girls	-0.54 [-0.56, -0.52]	0.71 [0.69, 0.72]	0.60 [0.58, 0.62]	0.33 [0.30, 0.36]
	Boys	-0.45 [-0.48, -0.42]	0.65 [0.63, 0.67]	0.53 [0.50, 0.56]	0.27 [0.23, 0.30]
**Executive function skills**					
Inhibition	*Total*	-0.41 [-0.43, -0.39]	0.66 [0.65, 0.67]	0.47 [0.46, 0.49]	0.28 [0.26, 0.30]
	Girls	-0.45 [-0.48, -0.43]	0.67 [0.65, 0.69]	0.51 [0.49, 0.54]	0.31 [0.28, 0.34]
	Boys	-0.36 [-0.39, -0.33]	0.65 [0.63, 0.67]	0.45 [0.42, 0.48]	0.23 [0.20, 0.27]
Self-Monitoring	*Total*	-0.41 [-0.43, -0.39]	0.59 [0.58, 0.61]	0.47 [0.45, 0.49]	0.28 [0.26, 0.30]
	Girls	-0.46 [-0.48, -0.43]	0.61 [0.59, 0.63]	0.51 [0.49, 0.53]	0.31 [0.28, 0.34]
	Boys	-0.38 [-0.41, -0.34]	0.58 [0.55, 0.60]	0.46 [0.43, 0.48]	0.23 [0.19, 0.26]
Shifting	*Total*	-0.50 [-0.51, -0.48]	0.65 [0.64, 0.66]	0.57 [0.55, 0.58]	0.29 [0.27, 0.32]
	Girls	-0.52 [-0.55, -0.50]	0.66 [0.65, 0.68]	0.59 [0.57, 0.61]	0.31 [0.28, 0.34]
	Boys	-0.44 [-0.47, -0.41]	0.62 [0.60, 0.65]	0.52 [0.50, 0.55]	0.25 [0.21, 0.28]
Emotional Control	*Total*	-0.51 [-0.53, -0.49]	0.69 [0.68, 0.70]	0.66 [0.65, 0.67]	0.38 [0.36, 0.40]
	Girls	-0.54 [-0.57, -0.52]	0.71 [0.69, 0.73]	0.67 [0.65, 0.69]	0.39 [0.36, 0.41]
	Boys	-0.41 [-0.44, -0.38]	0.65 [0.63, 0.67]	0.58 [0.55, 0.60]	0.31 [0.27, 0.34]
Task Completion	*Total*	-0.45 [-0.47, -0.43]	0.60 [0.59, 0.62]	0.49 [0.47, 0.51]	0.27 [0.25, 0.29]
	Girls	-0.48 [-0.50, -0.45]	0.62 [0.60, 0.64]	0.52 [0.50, 0.55]	0.28 [0.25, 0.31]
	Boys	-0.41 [-0.44, -0.37]	0.58 [0.56, 0.61]	0.46 [0.44, 0.49]	0.24 [0.20, 0.27]
Working Memory	*Total*	-0.46 [-0.48, -0.44]	0.64 [0.63, 0.66]	0.51 [0.50, 0.53]	0.28 [0.26, 0.30]
	Girls	-0.49 [-0.51, -0.47]	0.66 [0.64, 0.68]	0.54 [0.52, 0.56]	0.30 [0.27, 0.33]
	Boys	-0.42 [-0.45, -0.38]	0.61 [0.59, 0.64]	0.48 [0.45, 0.51]	0.24 [0.20, 0.27]
Planning	*Total*	-0.49 [-0.51, -0.47]	0.65 [0.64, 0.67]	0.55 [0.53, 0.57]	0.31 [0.29, 0.33]
	Girls	-0.52 [-0.54, -0.50]	0.67 [0.65, 0.69]	0.58 [0.56, 0.60]	0.33 [0.30, 0.36]
	Boys	-0.44 [-0.47, -0.41]	0.63 [0.61, 0.65]	0.53 [0.50, 0.55]	0.28 [0.24, 0.31]

Note. Please note that higher executive function scores mean greater difficulties.
WEMWBS=Warwick-Edinburgh Mental Well-Being Scale. SDQ=Strengths and Difficulties Questionnaire. CESD=Center for Epidemiological Studies-Depression. Suicidality=see **Supplement 1**.

Table S4. Spearman correlations for the cross-sectional (T1) relationships between emotional self-regulation and mental health.

	WEMWBS	SDQ	CESD	Suicidality
T1	r [95%CI]	r [95%CI]	r [95%CI]	r [95%CI]
**Shifting**				
2. I have trouble accepting a different way to solve a problem with things such as schoolwork, friends, or tasks.	-0.41 [-0.44, -0.39]	0.52 [0.50, 0.54]	0.48 [0.46, 0.50]	0.25 [0.23, 0.27]
11. I have trouble getting used to new situations (such as classes, groups, or friends).	-0.39 [-0.41, -0.36]	0.48 [0.46, 0.50]	0.44 [0.42, 0.46]	0.23 [0.21, 0.25]
17. I get suck on one topic or activity.	-0.33 [-0.35, -0.30]	0.44 [0.42, 0.46]	0.38 [0.36, 0.40]	0.20 [0.17, 0.22]
31. It bothers me when I have to deal with changes (such as routines, foods, or places).	-0.34 [-0.36, -0.32]	0.44 [0.41, 0.46]	0.39 [0.37, 0.42]	0.20 [0.17, 0.22]
40. I try the same approach to a problem over and over even when it does not work (I get stuck).	-0.32 [-0.35, -0.30]	0.43 [0.41, 0.45]	0.38 [0.35, 0.40]	0.20 [0.17, 0.22]
45. I have trouble thinking of a different way to solve a problem when I get stuck.	-0.36 [-0.38, -0.34]	0.45 [0.43, 0.47]	0.40 [0.38, 0.42]	0.22 [0.20, 0.24]
51. I have trouble changing from one activity to another.	-0.32 [-0.34, -0.30]	0.43 [0.41, 0.45]	0.36 [0.34, 0.38]	0.20 [0.17, 0.22]
53. I have difficulty coming up with different ways of solving a problem.	-0.36 [-0.38, -0.33]	0.44 [0.42, 0.46]	0.40 [0.38, 0.42]	0.21 [0.18, 0.23]
**Emotional control**				
6. I have angry outbursts.	-0.35 [-0.37, -0.33]	0.55 [0.53, 0.56]	0.44 [0.42, 0.46]	0.27 [0.25, 0.30]
14. I have outbursts for little reason.	-0.43 [-0.45, -0.41]	0.54 [0.52, 0.55]	0.56 [0.55, 0.58]	0.33 [0.31, 0.36]
22. I get upset over small events.	-0.41 [-0.43, -0.39]	0.50 [0.48, 0.52]	0.51 [0.49, 0.53]	0.29 [0.27, 0.31]
27. I overreact.	-0.32 [-0.34, -0.30]	0.46 [0.44, 0.48]	0.40 [0.38, 0.42]	0.22 [0.20, 0.24]
34. I am easily overwhelmed.	-0.39 [-0.41, -0.37]	0.51 [0.49, 0.52]	0.49 [0.47, 0.51]	0.27 [0.24, 0.29]
43. My eyes fill with tears quickly over little things.	-0.37 [-0.39, -0.35]	0.47 [0.45, 0.49]	0.49 [0.48, 0.51]	0.27 [0.25, 0.30]

Note. Please note that higher shifting and emotional control scores mean greater difficulties.
WEMWBS=Warwick-Edinburgh Mental Well-Being Scale. SDQ=Strengths and Difficulties Questionnaire. CESD=Center for Epidemiological Studies-Depression. Suicidality=see **Supplement 1**.

Figure S2. Interaction plots for the univariable analyses based on the three-level random intercept model

Self-Reported Executive Function

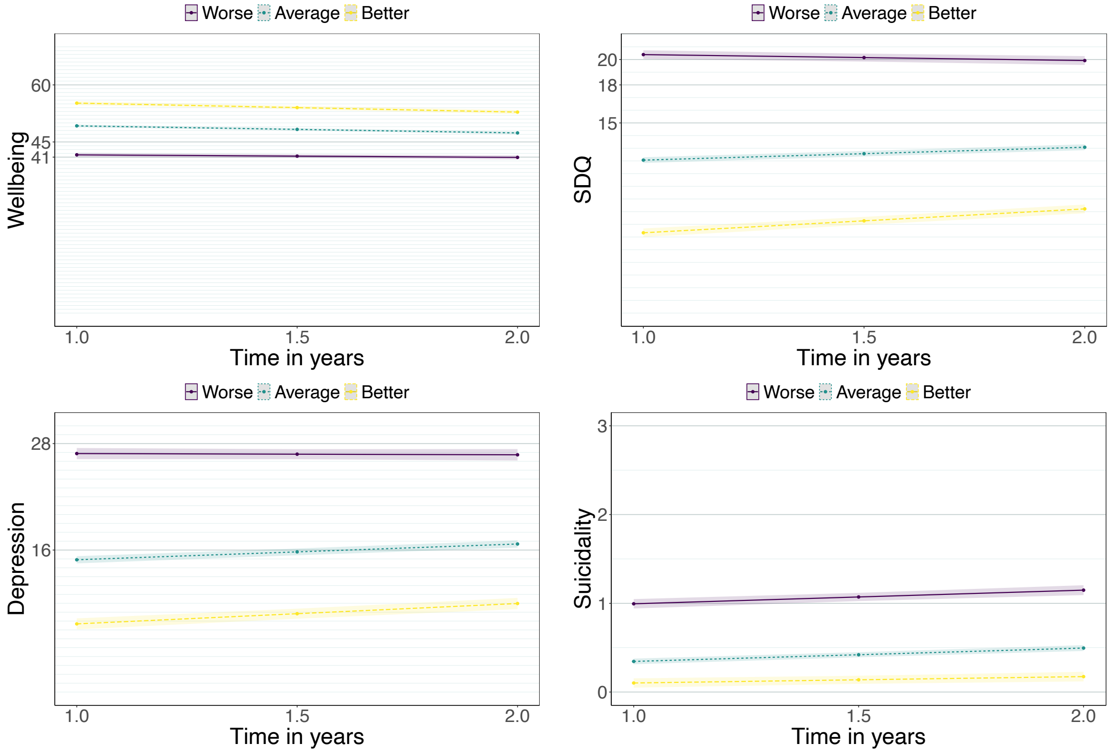

Note. Worse: +1 standard deviation, Average: Mean, Better: -1 standard deviation.
Cut-off scores are based on the official scoring guidelines.
Well-being: probable mental health difficulties (0-40); possible mental health difficulties (41-44); average mental well-being (45-59); high well-being (60-70; Tennant et al., 2007; Warwick Medical School, 2021).
Social-emotional-behavioral difficulties [SDQ]: normal (0–14); borderline (15–17); high (18–19); very high (20–40; Youth in Mind, 2016).
Depression: low (0–15); at risk of depression (16–27); caseness (28–60; Radloff, 1977; Radloff, 1991).
Suicidality: cut-off values: no suicidal risk (0); live not worth living (1); self-harm thoughts (2); self-harm behaviors (3).

Self-Reported Behavioural Self-Regulation

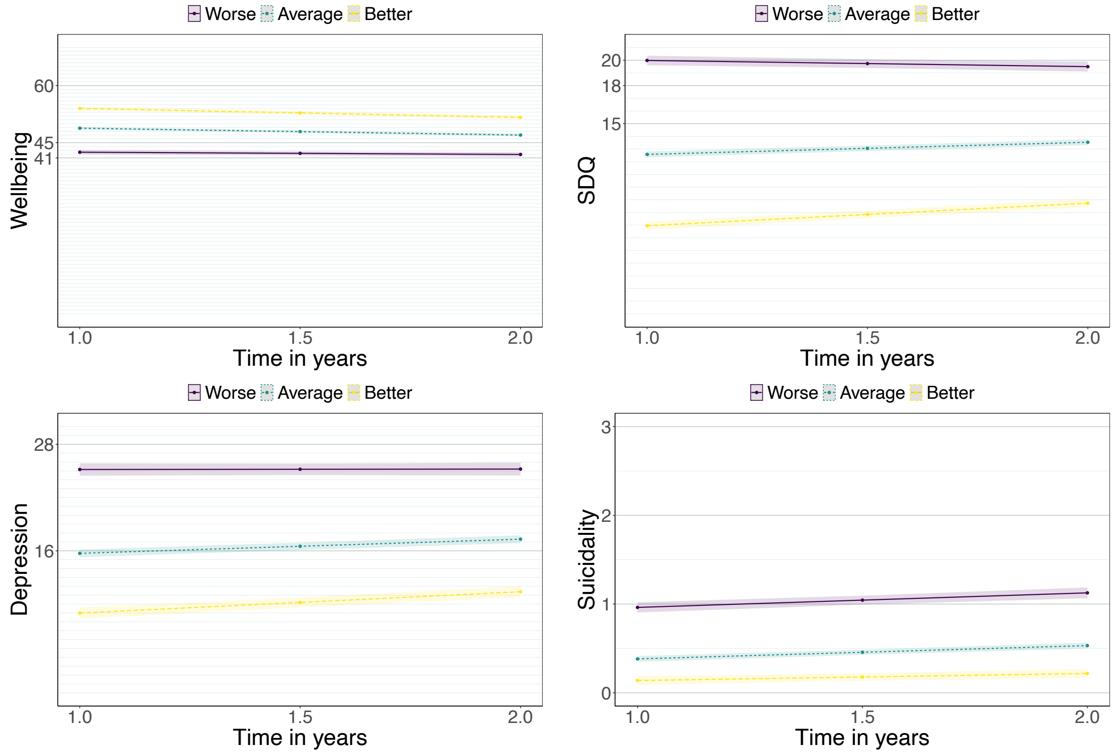

Self-Reported Emotional Self-Regulation

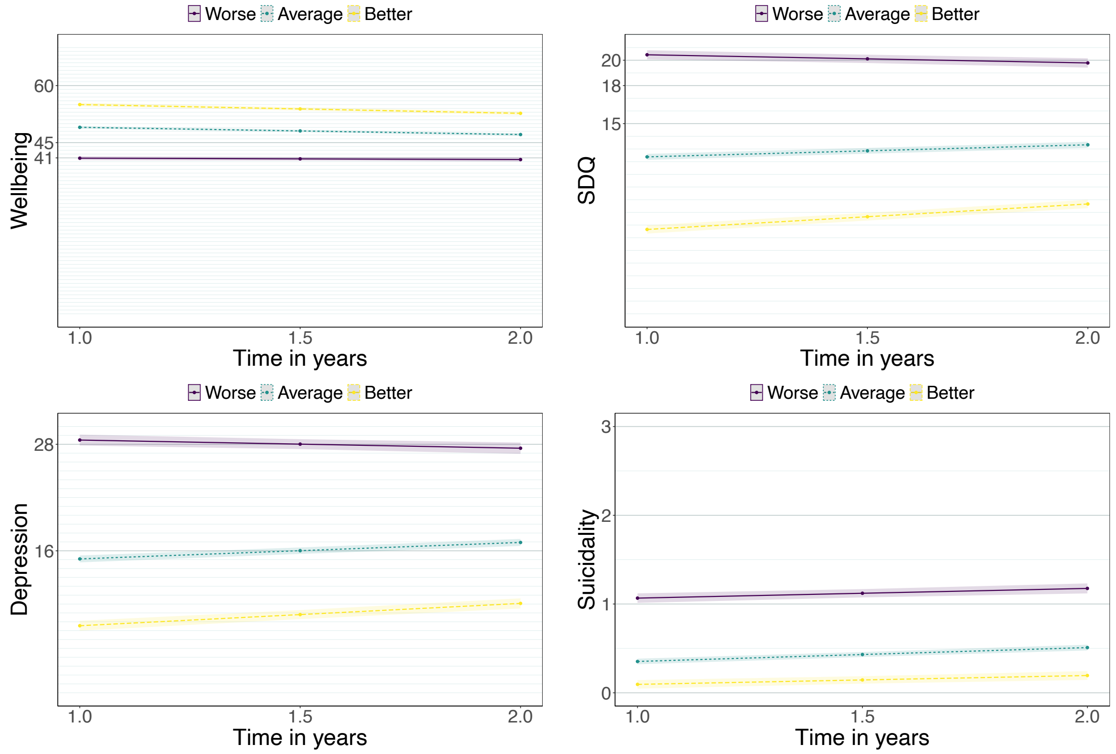

Note. Worse: +1 standard deviation, Average: Mean, Better: -1 standard deviation.
Cut-off scores are based on the official scoring guidelines.
Well-being: probable mental health difficulties (0-40); possible mental health difficulties (41-44); average mental well-being (45-59); high well-being (60-70; Tennant et al., 2007; Warwick Medical School, 2021).
Social-emotional-behavioral difficulties [SDQ]: normal (0–14); borderline (15–17); high (18–19); very high (20–40; Youth in Mind, 2016).
Depression: low (0–15); at risk of depression (16–27); caseness (28–60; Radloff, 1977; Radloff, 1991).
Suicidality: cut-off values: no suicidal risk (0); live not worth living (1); self-harm thoughts (2); self-harm behaviors (3).

Self-Reported Cognitive Self-Regulation

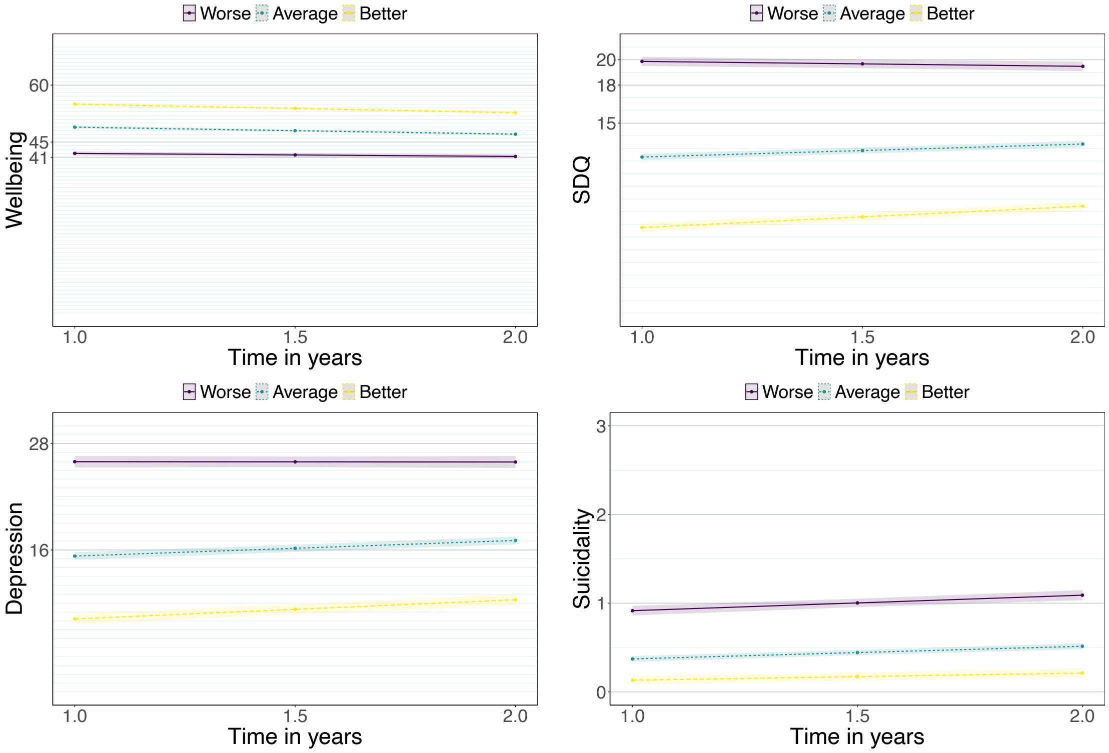

Note. Worse: +1 standard deviation, Average: Mean, Better: -1 standard deviation.
Cut-off scores are based on the official scoring guidelines.
Well-being: probable mental health difficulties (0-40); possible mental health difficulties (41-44); average mental well-being (45-59); high well-being (60-70; Tennant et al., 2007; Warwick Medical School, 2021).
Social-emotional-behavioral difficulties [SDQ]: normal (0–14); borderline (15–17); high (18–19); very high (20–40; Youth in Mind, 2016).
Depression: low (0–15); at risk of depression (16–27); caseness (28–60; Radloff, 1977; Radloff, 1991).
Suicidality: cut-off values: no suicidal risk (0); live not worth living (1); self-harm thoughts (2); self-harm behaviors (3).

Self-Reported Inhibition Skills

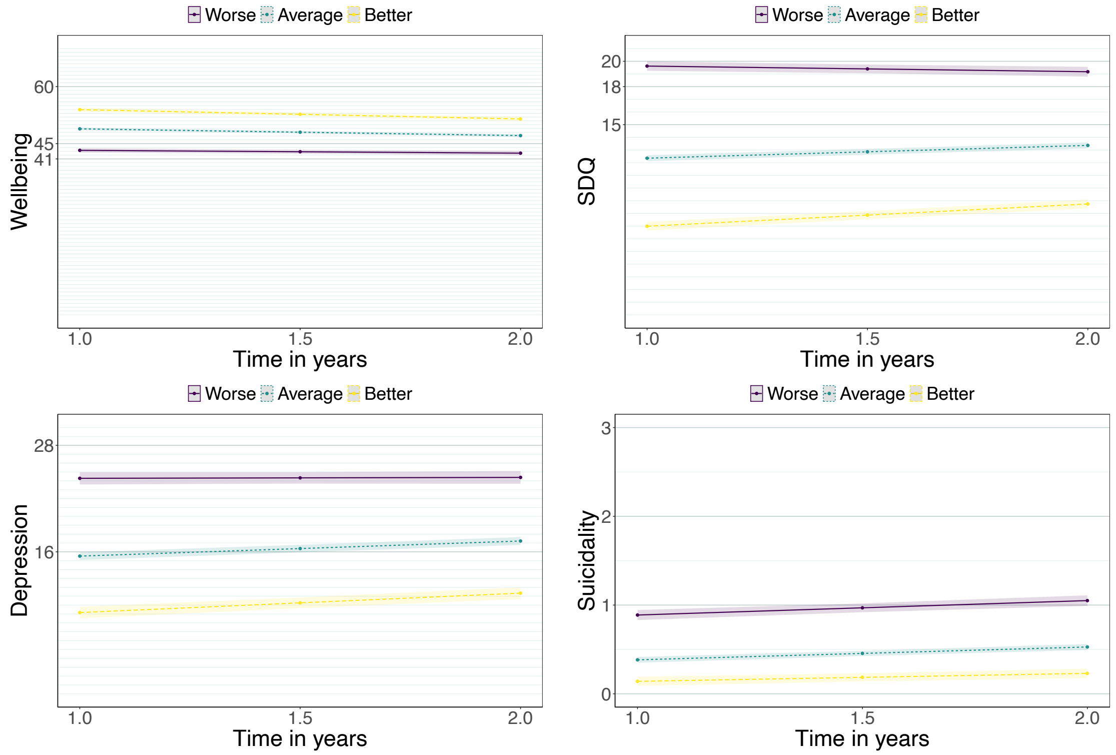

Self-Reported Self-Monitoring Skills

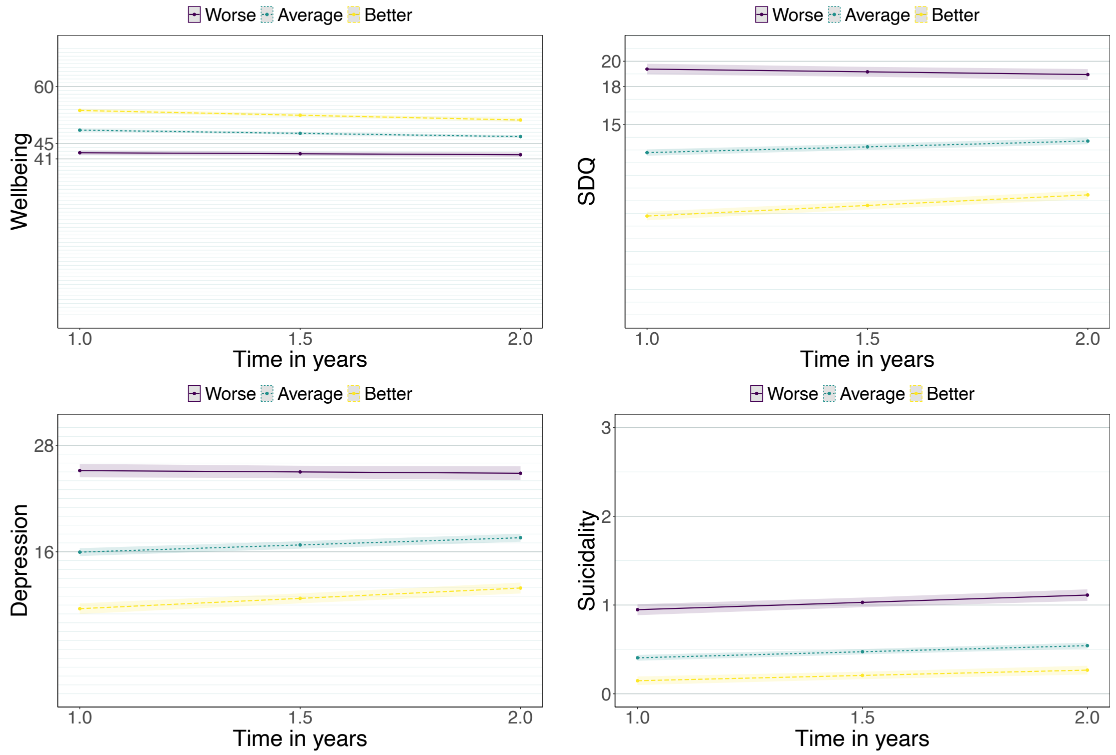

Note. Worse: +1 standard deviation, Average: Mean, Better: -1 standard deviation.
Cut-off scores are based on the official scoring guidelines.
Well-being: probable mental health difficulties (0-40); possible mental health difficulties (41-44); average mental well-being (45-59); high well-being (60-70; Tennant et al., 2007; Warwick Medical School, 2021).
Social-emotional-behavioral difficulties [SDQ]: normal (0–14); borderline (15–17); high (18–19); very high (20–40; Youth in Mind, 2016).
Depression: low (0–15); at risk of depression (16–27); caseness (28–60; Radloff, 1977; Radloff, 1991).
Suicidality: cut-off values: no suicidal risk (0); live not worth living (1); self-harm thoughts (2); self-harm behaviors (3).

Self-Reported Shifting Skills

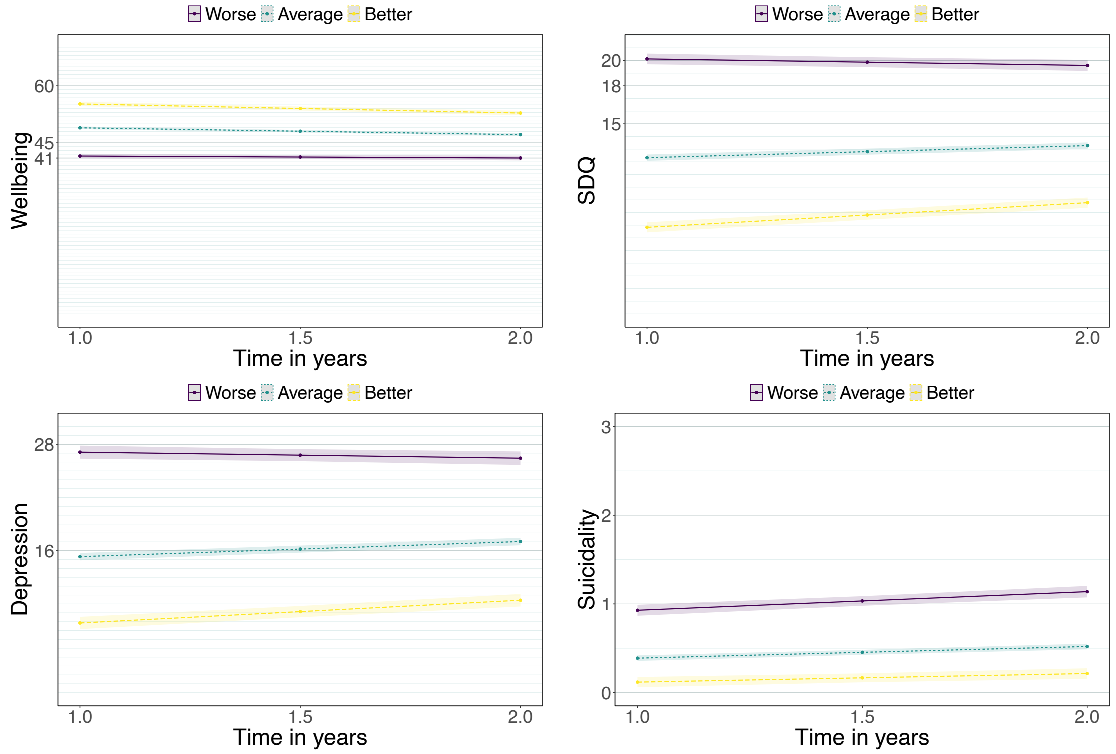

Self-Reported Emotional Control Skills

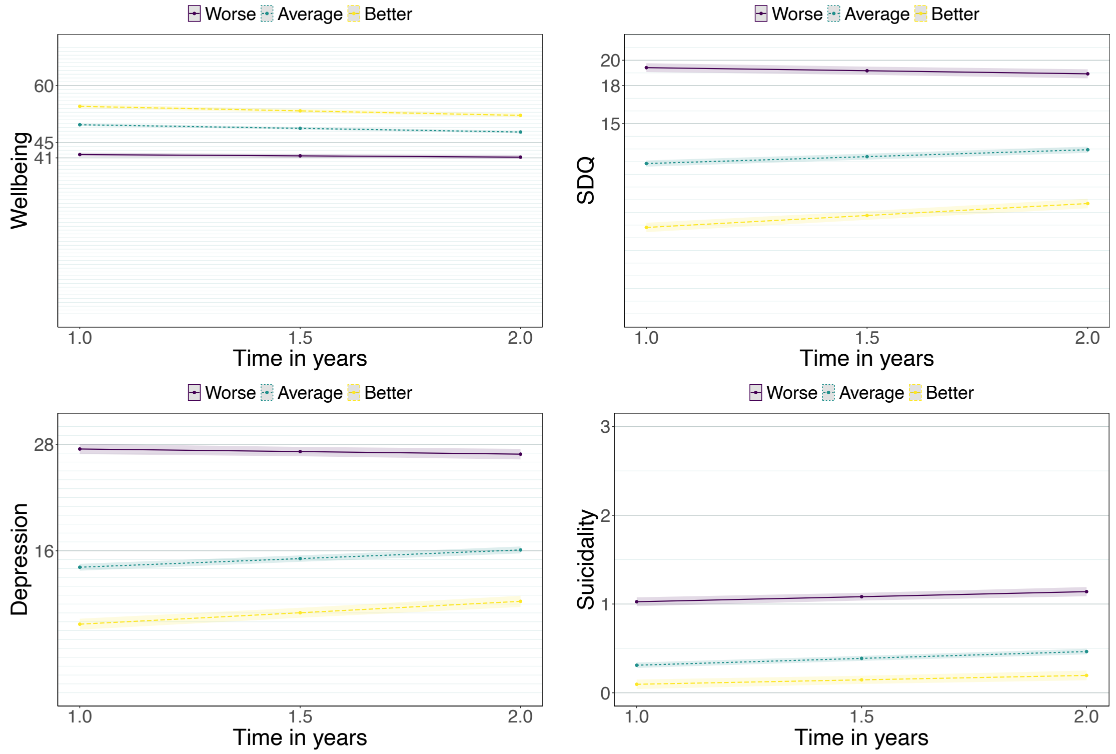

Note. Worse: +1 standard deviation, Average: Mean, Better: -1 standard deviation.
Cut-off scores are based on the official scoring guidelines.
Well-being: probable mental health difficulties (0-40); possible mental health difficulties (41-44); average mental well-being (45-59); high well-being (60-70; Tennant et al., 2007; Warwick Medical School, 2021).
Social-emotional-behavioral difficulties [SDQ]: normal (0–14); borderline (15–17); high (18–19); very high (20–40; Youth in Mind, 2016).
Depression: low (0–15); at risk of depression (16–27); caseness (28–60; Radloff, 1977; Radloff, 1991).
Suicidality: cut-off values: no suicidal risk (0); live not worth living (1); self-harm thoughts (2); self-harm behaviors (3).

Self-Reported Task Completion Skills

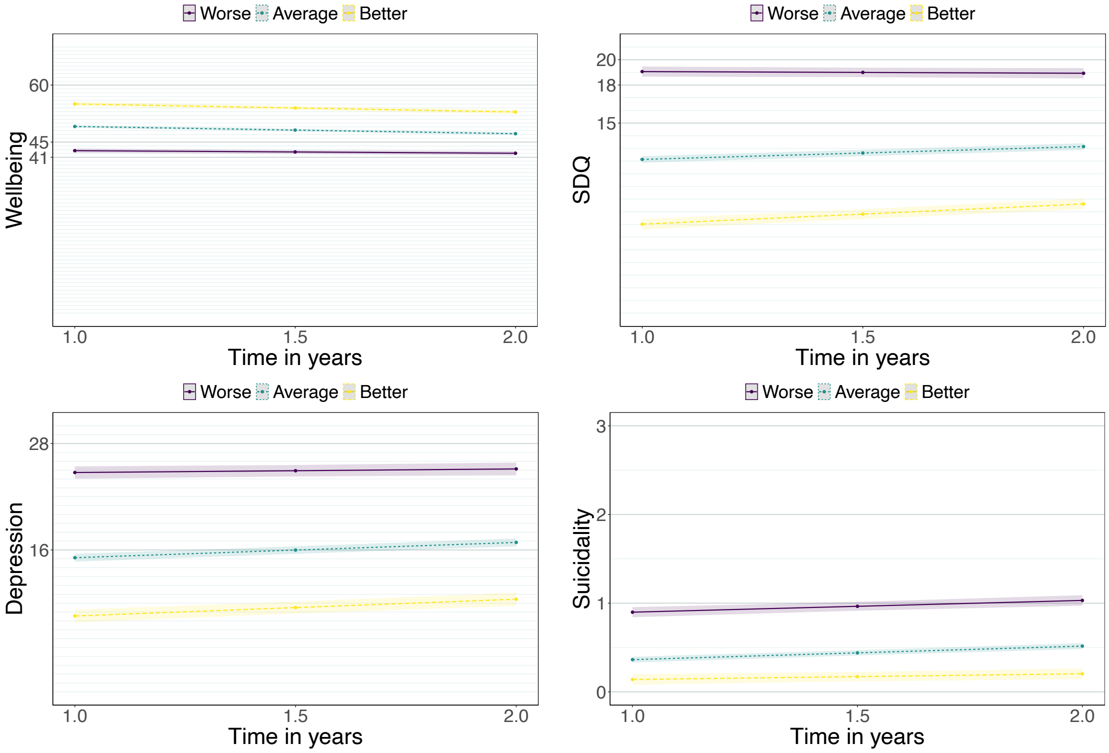

Self-Reported Working Memory Skills

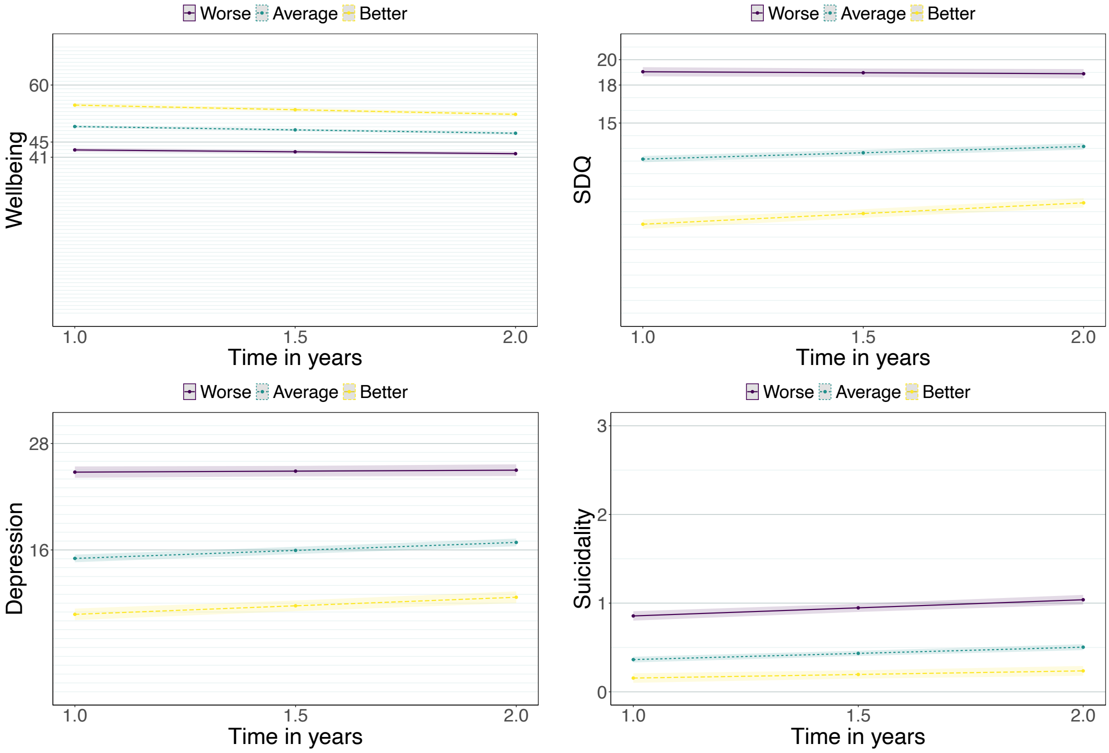

Note. Worse: +1 standard deviation, Average: Mean, Better: -1 standard deviation.
Cut-off scores are based on the official scoring guidelines.
Well-being: probable mental health difficulties (0-40); possible mental health difficulties (41-44); average mental well-being (45-59); high well-being (60-70; Tennant et al., 2007; Warwick Medical School, 2021).
Social-emotional-behavioral difficulties [SDQ]: normal (0–14); borderline (15–17); high (18–19); very high (20–40; Youth in Mind, 2016).
Depression: low (0–15); at risk of depression (16–27); caseness (28–60; Radloff, 1977; Radloff, 1991).
Suicidality: cut-off values: no suicidal risk (0); live not worth living (1); self-harm thoughts (2); self-harm behaviors (3).

Self-Reported Planning Skills

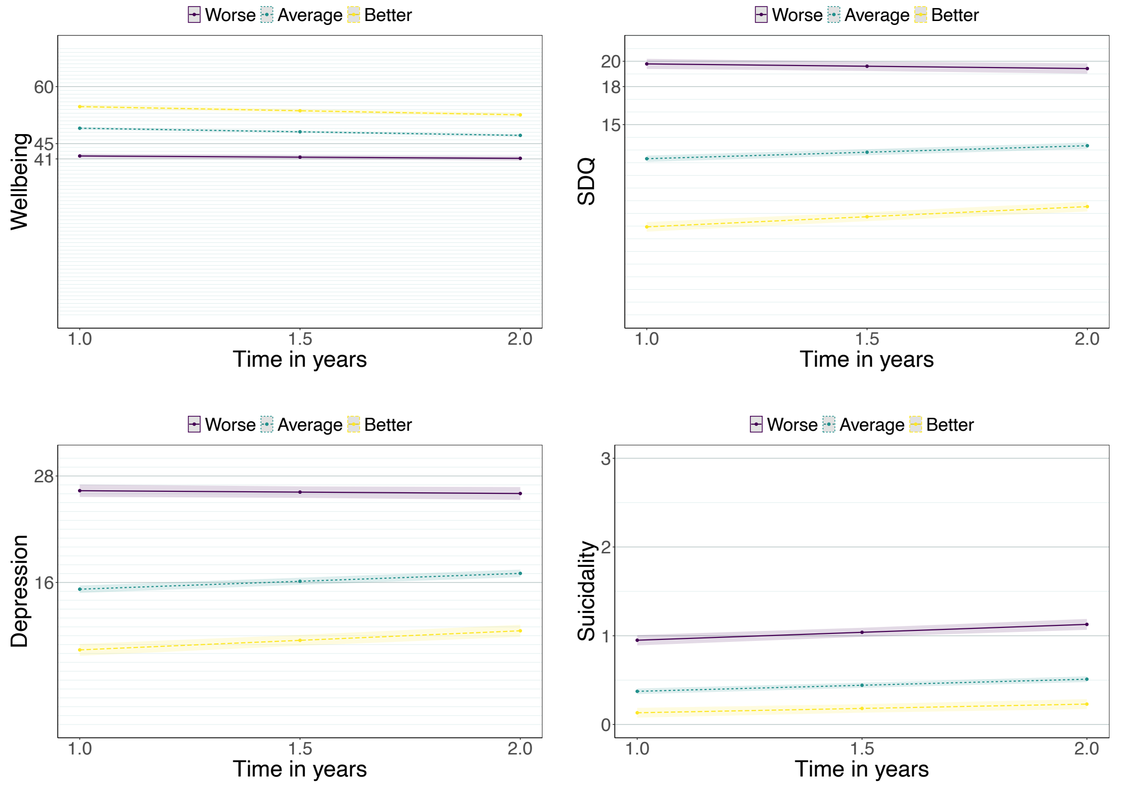

Note. Worse: +1 standard deviation, Average: Mean, Better: -1 standard deviation.
Cut-off scores are based on the official scoring guidelines.
Well-being: probable mental health difficulties (0-40); possible mental health difficulties (41-44); average mental well-being (45-59); high well-being (60-70; Tennant et al., 2007; Warwick Medical School, 2021).
Social-emotional-behavioral difficulties [SDQ]: normal (0–14); borderline (15–17); high (18–19); very high (20–40; Youth in Mind, 2016).
Depression: low (0–15); at risk of depression (16–27); caseness (28–60; Radloff, 1977; Radloff, 1991).
Suicidality: cut-off values: no suicidal risk (0); live not worth living (1); self-harm thoughts (2); self-harm behaviors (3).

Table S5. Univariable analyses (unadjusted), based on the three-level random intercept model.

	Well-Being				Social-Emotional-Behavioral Difficulties				Depression				Suicidality			
	**B**	**95% CI**	**p**	**LRT p**	**B**	**95% CI**	**p**	**LRT p**	**B**	**95% CI**	**p**	**LRT p**	**B**	**95% CI**	**p**	**LRT p**
**Executive function (total [EF])**																
EF	-0.23	[-0.24, -0.22]	**< .001**	**< .001**	0.24	[0.23, 0.24]	**< .001**	**< .001**	0.33	[0.32, 0.34]	**< .001**	**< .001**	0.02	[0.01, 0.02]	**< .001**	0.084
Time(T2)*EF	0.02	[0.01, 0.03]	**< .001**		-0.02	[-0.03, -0.02]	**< .001**		-0.03	[-0.05, -0.02]	**< .001**		NA	NA	NA	
Time(T3)*EF	0.03	[0.02, 0.04]	**< .001**		-0.04	[-0.04, -0.03]	**< .001**		-0.05	[-0.06, -0.04]	**< .001**		NA	NA	NA	
**Self-regulation**																
Behavioral	-0.70	[-0.74, -0.66]	**< .001**	**< .001**	0.79	[0.77, 0.81]	**< .001**	**< .001**	0.99	[0.95, 1.04]	**< .001**	**< .001**	0.05	[0.05, 0.05]	**< .001**	0.087
Time(T2)*Behavioral	0.09	[0.05, 0.12]	**< .001**		-0.08	[-0.11, -0.06]	**< .001**		-0.11	[-0.15, -0.07]	**< .001**		NA	NA	NA	
Time(T3)*Behavioral	0.12	[0.08, 0.15]	**< .001**		-0.13	[-0.15, -0.11]	**< .001**		-0.15	[-0.20, -0.11]	**< .001**		NA	NA	NA	
Emotional	-0.80	[-0.83, -0.77]	**< .001**	**< .001**	0.78	[0.76, 0.80]	**< .001**	**< .001**	1.20	[1.16, 1.23]	**< .001**	**< .001**	0.05	[0.05, 0.06]	**< .001**	0.453
Time(T2)*Emotional	0.09	[0.05, 0.12]	**< .001**		-0.09	[-0.11, -0.07]	**< .001**		-0.15	[-0.19, -0.12]	**< .001**		NA	NA	NA	
Time(T3)*Emotional	0.11	[0.07, 0.15]	**< .001**		-0.14	[-0.16, -0.12]	**< .001**		-0.19	[-0.23, -0.15]	**< .001**		NA	NA	NA	
Cognitive	-0.43	[-0.45, -0.41]	**< .001**	**< .001**	0.43	[0.42, 0.45]	**< .001**	**< .001**	0.60	[0.57, 0.62]	**< .001**	**< .001**	0.03	[0.03, 0.03]	**< .001**	0.054
Time(T2)*Cognitive	0.04	[0.02, 0.06]	**< .001**		-0.04	[-0.05, -0.03]	**< .001**		-0.05	[-0.08, -0.03]	**< .001**		NA	NA	NA	
Time(T3)*Cognitive	0.05	[0.03, 0.07]	**< .001**		-0.07	[-0.08, -0.05]	**< .001**		-0.07	[-0.10, -0.05]	**< .001**		NA	NA	NA	
**Executive function skills**																
Inhibition	-1.02	[-1.08, -0.96]	**< .001**	**< .001**	1.21	[1.18, 1.25]	**< .001**	**< .001**	1.46	[1.39, 1.54]	**< .001**	**< .001**	0.07	[0.07, 0.06]	**< .001¥**	**0.041**
Time(T2)*Inhibition	0.12	[0.07, 0.18]	**< .001**		-0.13	[-0.16, -0.09]	**< .001**		-0.16	[-0.23, -0.10]	**< .001**		0.00	[-0.01, 0.01]	0.221¥	
Time(T3)*Inhibition	0.16	[0.10, 0.22]	**< .001**		-0.20	[-0.24, -0.17]	**< .001**		-0.21	[-0.28, -0.15]	**< .001**		0.00	[0.01, 0.02]	**0.011¥**	
Self-Monitoring	-1.54	[-1.63, -1.45]	**< .001**	**< .001**	1.61	[1.55, 1.67]	**< .001**	**< .001**	2.15	[2.05, 2.26]	**< .001**	**< .001**	0.11	[0.10, 0.12]	**< .001**	0.390
Time(T2)*Monitoring	0.21	[0.12, 0.29]	**< .001**		-0.18	[-0.23, -0.13]	**< .001**		-0.25	[-0.35, -0.16]	**< .001**		NA	NA	NA	
Time(T3)*Monitoring	0.27	[0.19, 0.36]	**< .001**		-0.28	[-0.33, -0.23]	**< .001**		-0.37	[-0.47, -0.27]	**< .001**		NA	NA	NA	
Shifting	-1.25	[-1.31, -1.19]	**< .001**	**< .001**	1.21	[1.17, 1.24]	**< .001**	**< .001**	1.76	[1.69, 1.83]	**< .001**	**< .001**	0.08	[0.07, 0.08]	**< .001**	0.310
Time(T2)*Shifting	0.14	[0.09, 0.20]	**< .001**		-0.13	[-0.17, -0.10]	**< .001**		-0.21	[-0.28, -0.15]	**< .001**		NA	NA	NA	
Time(T3)*Shifting	0.18	[0.12, 0.23]	**< .001**		-0.21	[-0.25, -0.18]	**< .001**		-0.28	[-0.35, -0.22]	**< .001**		NA	NA	NA	
Emotional Control	-1.46	[-1.53, -1.40]	**< .001**	**< .001**	1.45	[1.40, 1.49]	**< .001**	**< .001**	2.32	[2.24, 2.39]	**< .001**	**< .001**	0.11	[0.10, 0.12]	**< .001**	0.502
Time(T2)*EmoContr	0.15	[0.09, 0.22]	**< .001**		-0.19	[-0.23, -0.15]	**< .001**		-0.30	[-0.38, -0.23]	**< .001**		NA	NA	NA	
Time(T3)*EmoContr	0.20	[0.14, 0.27]	**< .001**		-0.27	[-0.31, -0.23]	**< .001**		-0.37	[-0.45, -0.30]	**< .001**		NA	NA	NA	
Task Completion	-1.20	[-1.26, -1.14]	**< .001**	**< .001**	1.19	[1.15, 1.24]	**< .001**	**< .001**	1.63	[1.55, 1.71]	**< .001**	**< .001**	0.08	[0.07, 0.08]	**< .001¥**	0.316
Time(T2)*Task	0.12	[0.06, 0.18]	**< .001**		-0.11	[-0.15, -0.08]	**< .001**		-0.15	[-0.22, -0.08]	**< .001**		NA	NA	NA	
Time(T3)*Task	0.17	[0.11, 0.24]	**< .001**		-0.18	[-0.21, -0.14]	**< .001**		-0.20	[-0.27, -0.13]	**< .001**		NA	NA	NA	
Working Memory	-1.12	[-1.18, -1.07]	**< .001**	**< .001**	1.15	[1.12, 1.19]	**< .001**	**< .001**	1.55	[1.48, 1.61]	**< .001**	**< .001**	0.07	[0.06, 0.07]	**< .001**	**0.006**
Time(T2)*WM	0.10	[0.05, 0.16]	**< .001**		-0.10	[-0.14, -0.07]	**< .001**		-0.12	[-0.19, -0.06]	**< .001**		0.00	[0.00, 0.01]	**0.024**	
Time(T3)*WM	0.13	[0.08, 0.19]	**< .001**		-0.18	[-0.21, -0.14]	**< .001**		-0.17	[-0.23, -0.10]	**< .001**		0.01	[0.00, 0.02]	**0.002**	
Planning	-1.02	[-1.07, -0.98]	**< .001**	**< .001**	1.01	[0.98, 1.04]	**< .001**	**< .001**	1.43	[1.37, 1.49]	**< .001**	**< .001**	0.07	[0.06, 0.07]	**< .001**	0.152
Time(T2)*Planning	0.09	[0.04, 0.13]	**< .001**		-0.09	[-0.12, -0.06]	**< .001**		-0.13	[-0.19, -0.08]	**< .001**		NA	NA	NA	
Time(T3)*Planning	0.12	[0.07, 0.17]	**< .001**		-0.16	[-0.18, -0.13]	**< .001**		-0.20	[-0.26, -0.15]	**< .001**		NA	NA	NA	

Note: We present the unstandardized regression coefficients to show the actual change in the outcome measure associated with a one-unit change in the predictor. Executive function, the underpinning subdimensions and skills, were cluster-mean centered and treated as time-constant (i.e., analyzed only at T1). Please note that higher executive function scores mean greater difficulties. Students are nested within schools. LRT=likelihood ratio test to evaluate whether the time*predictor interaction effect needs to be included in the model. NA=only the main effect of time, but not the time*predictor interaction term, was included. T1 is the reference. ¥To address convergence problems, this model was estimated with the "bobyqa" optimizer for a quadratic approximation and full maximum likelihood estimation.

Table S6. Univariable analyses (unadjusted) for girls, based on the three-level random intercept model.

	Well-Being				Social-Emotional-Behavioral Difficulties				Depression				Suicidality			
	**B**	**95% CI**	**p**	**LRT p**	**B**	**95% CI**	**p**	**LRT p**	**B**	**95% CI**	**p**	**LRT p**	**B**	**95% CI**	**p**	**LRT p**
**Executive function (total [EF])**																
EF	-0.25	[-0.27, -0.24]	**< .001**	**< .001**	0.25	[0.24, 0.26]	**< .001**	**< .001**	0.37	[0.35, 0.39]	**< .001**	**< .001**	0.02	[0.02, 0.02]	**< .001**	0.127
Time(T2)*EF	0.03	[0.02, 0.05]	**< .001**		-0.03	[-0.03, -0.02]	**< .001**		-0.05	[-0.06, -0.03]	**< .001**		NA	NA	NA	
Time(T3)*EF	0.04	[0.03, 0.05]	**< .001**		-0.04	[-0.05, -0.03]	**< .001**		-0.06	[-0.07, -0.04]	**< .001**		NA	NA	NA	
**Self-regulation**																
Behavioral	-0.78	[-0.83, -0.73]	**< .001**	**< .001**	0.82	[0.79, 0.86]	**< .001**	**< .001**	1.14	[1.08, 1.20]	**< .001**¥	**< .001**	0.06	[0.06, 0.07]	**< .001**	0.351
Time(T2)*Behavioral	0.11	[0.06, 0.15]	**< .001**		-0.09	[-0.12, -0.06]	**< .001**		-0.15	[-0.21, -0.09]	**< .001**¥		NA	NA	NA	
Time(T3)*Behavioral	0.12	[0.07, 0.17]	**< .001**		-0.14	[-0.17, -0.11]	**< .001**		-0.19	[-0.25, -0.13]	**< .001**¥		NA	NA	NA	
Emotional	-0.84	[-0.88, -0.80]	**< .001**	**< .001**	0.80	[0.77, 0.83]	**< .001**	**< .001**	1.28	[1.23, 1.33]	**< .001**	**< .001**	0.06	[0.06, 0.06]	**< .001**	0.447
Time(T2)*Emotional	0.13	[0.08, 0.17]	**< .001**		-0.11	[-0.14, -0.09]	**< .001**		-0.21	[-0.26, -0.15]	**< .001**		NA	NA	NA	
Time(T3)*Emotional	0.15	[0.10, 0.19]	**< .001**		-0.16	[-0.18, -0.13]	**< .001**		-0.24	[-0.29, -0.18]	**< .001**		NA	NA	NA	
Cognitive	-0.47	[-0.50, -0.45]	**< .001**	**< .001**	0.46	[0.44, 0.47]	**< .001**	**< .001**	0.67	[0.64, 0.71]	**< .001**	**< .001**	0.03	[0.03, 0.03]	**< .001**	**0.043**
Time(T2)*Cognitive	0.06	[0.03, 0.08]	**< .001**		-0.04	[-0.05, -0.02]	**< .001**		-0.07	[-0.11, -0.04]	**< .001**		0.00	[0.00, 0.01]	**0.026**	
Time(T3)*Cognitive	0.07	[0.04, 0.09]	**< .001**		-0.07	[-0.08, -0.05]	**< .001**		-0.09	[-0.13, -0.06]	**< .001**		0.00	[0.00, 0.01]	**0.040**	
**Executive function skills**																
Inhibition	-1.14	[-1.22, -1.06]	**< .001**	**< .001**	1.26	[1.21, 1.31]	**< .001**	**< .001**	1.68	[1.58, 1.78]	**< .001**	**< .001**	0.09	[0.08, 0.10]	**< .001**	0.239
Time(T2)*Inhibition	0.15	[0.07, 0.23]	**< .001**		-0.14	[-0.19, -0.10]	**< .001**		-0.22	[-0.31, -0.12]	**< .001**		NA	NA	NA	
Time(T3)*Inhibition	0.16	[0.08, 0.24]	**< .001**		-0.21	[-0.26, -0.17]	**< .001**		-0.27	[-0.36, -0.17]	**< .001**		NA	NA	NA	
Self-Monitoring	-1.71	[-1.82, -1.59]	**< .001**	**< .001**	1.68	[1.60, 1.75]	**< .001**	**< .001**	2.46	[2.31, 2.60]	**< .001**	**< .001**	0.13	[0.12, 0.14]	**< .001**	0.695
Time(T2)*Monitoring	0.24	[0.13, 0.35]	**< .001**		-0.20	[-0.26, -0.13]	**< .001**		-0.34	[-0.48, -0.21]	**< .001**		NA	NA	NA	
Time(T3)*Monitoring	0.31	[0.20, 0.43]	**< .001**		-0.30	[-0.37, -0.23]	**< .001**		-0.45	[-0.59, -0.32]	**< .001**		NA	NA	NA	
Shifting	-1.31	[-1.39, -1.23]	**< .001**	**< .001**	1.23	[1.18, 1.28]	**< .001**¥	**< .001**	1.90	[1.81, 2.00]	**< .001**	**< .001**	0.09	[0.08, 0.09]	**< .001**	0.258
Time(T2)*Shifting	0.18	[0.11, 0.26]	**< .001**		-0.15	[-0.19, -0.10]	**< .001**¥		-0.29	[-0.39, -0.20]	**< .001**		NA	NA	NA	
Time(T3)*Shifting	0.22	[0.14, 0.30]	**< .001**		-0.23	[-0.27, -0.18]	**< .001**¥		-0.34	[-0.43, -0.24]	**< .001**		NA	NA	NA	
Emotional Control	-1.51	[-1.59, -1.43]	**< .001**	**< .001**	1.46	[1.41, 1.51]	**< .001**	**< .001**	2.41	[2.31, 2.51]	**< .001**	**< .001**	0.12	[0.11, 0.12]	**< .001**	0.672
Time(T2)*EmoContr	0.26	[0.17, 0.34]	**< .001**		-0.24	[-0.29, -0.19]	**< .001**		-0.40	[-0.50, -0.30]	**< .001**		NA	NA	NA	
Time(T3)*EmoContr	0.28	[0.20, 0.37]	**< .001**		-0.31	[-0.35, -0.26]	**< .001**		-0.47	[-0.57, -0.37]	**< .001**		NA	NA	NA	
Task Completion	-1.30	[-1.38, -1.22]	**< .001**¥	**< .001**	1.24	[1.19, 1.30]	**< .001**	**< .001**	1.83	[1.72, 1.93]	**< .001**	**< .001**	0.09	[0.08, 0.10]	**< .001**¥	0.270
Time(T2)*Task	0.16	[0.08, 0.25]	**< .001**¥		-0.11	[-0.16, -0.06]	**< .001**		-0.22	[-0.32, -0.12]	**< .001**		NA	NA	NA	
Time(T3)*Task	0.20	[0.12, 0.28]	**< .001**¥		-0.18	[-0.23, -0.13]	**< .001**		-0.24	[-0.34, -0.13]	**< .001**		NA	NA	NA	
Working Memory	-1.20	[-1.28, -1.13]	**< .001**	**< .001**	1.20	[1.15, 1.25]	**< .001**	**< .001**	1.70	[1.61, 1.80]	**< .001**¥	**< .001**	0.08	[0.07, 0.09]	**< .001**	**0.008**
Time(T2)*WM	0.15	[0.07, 0.22]	**< .001**		-0.10	[-0.14, -0.06]	**< .001**		-0.16	[-0.25, -0.07]	**< .001**¥		0.01	[0.00, 0.02]	**0.007**	
Time(T3)*WM	0.16	[0.08, 0.23]	**< .001**		-0.17	[-0.22, -0.13]	**< .001**		-0.20	[-0.29, -0.11]	**< .001**¥		0.01	[0.00, 0.02]	**0.009**	
Planning	-1.11	[-1.17, -1.04]	**< .001**	**< .001**	1.05	[1.01, 1.09]	**< .001**	**< .001**	1.59	[1.51, 1.67]	**< .001**	**< .001**	0.08	[0.07, 0.09]	**< .001**	0.109
Time(T2)*Planning	0.13	[0.06, 0.19]	**< .001**		-0.09	[-0.13, -0.05]	**< .001**		-0.19	[-0.27, -0.11]	**< .001**		NA	NA	NA	
Time(T3)*Planning	0.16	[0.10, 0.23]	**< .001**		-0.16	[-0.20, -0.12]	**< .001**		-0.26	[-0.34, -0.18]	**< .001**		NA	NA	NA	

Note: We present the unstandardized regression coefficients to show the actual change in the outcome measure associated with a one-unit change in the predictor. Executive function, the underpinning subdimensions and skills, were cluster-mean centered and treated as time-constant (i.e., analyzed only at T1). Please note that higher executive function scores mean greater difficulties. Students are nested within schools. LRT=likelihood ratio test to evaluate whether the time*predictor interaction effect needs to be included in the model. NA=only the main effect of time, but not the time*predictor interaction term, was included. T1 is the reference. ¥To address convergence problems, this model was estimated with the "bobyqa" optimizer for a quadratic approximation and full maximum likelihood estimation.

Table S7. Univariable analyses (unadjusted) for boys, based on the three-level random intercept model.

	Well-Being				Social-Emotional-Behavioral Difficulties				Depression				Suicidality			
	**B**	**95% CI**	**p**	**LRT p**	**B**	**95% CI**	**p**	**LRT p**	**B**	**95% CI**	**p**	**LRT p**	**B**	**95% CI**	**p**	**LRT p**
**Executive function (total [EF])**																
EF	-0.19	[-0.20, -0.17]	**< .001**	**0.008**	0.22	[0.21, 0.23]	**< .001**	**< .001**	0.25	[0.24, 0.27]	**< .001**	**< .001**	0.01	[0.01, 0.01]	**< .001**	0.711
Time(T2)*EF	0.02	[0.00, 0.03]	**0.032**		-0.02	[-0.03, -0.01]	**< .001**		-0.02	[-0.04, 0.00]	**0.020**		NA	NA	NA	
Time(T3)*EF	0.02	[0.01, 0.04]	**0.003**		-0.04	[-0.05, -0.03]	**< .001**		-0.04	[-0.05, -0.02]	**< .001**		NA	NA	NA	
**Self-regulation**																
Behavioral	-0.58	[-0.64, -0.52]	**< .001**	**0.002**	0.73	[0.70, 0.77]	**< .001**	**< .001**	0.78	[0.72, 0.84]	**< .001**	**0.002**	0.03	[0.03, 0.04]	**< .001**	0.230
Time(T2)*Behavioral	0.07	[0.01, 0.13]	**0.021**		-0.07	[-0.11, -0.04]	**< .001**		-0.06	[-0.12, 0.00]	0.066		NA	NA	NA	
Time(T3)*Behavioral	0.10	[0.04, 0.16]	**< .001**		-0.13	[-0.17, -0.10]	**< .001**		-0.11	[-0.17, -0.05]	**< .001**		NA	NA	NA	
Emotional	-0.68	[-0.73, -0.62]	**< .001**	**0.001**	0.75	[0.72, 0.79]	**< .001**	**< .001**	0.96	[0.90, 1.01]	**< .001**	**< .001**	0.04	[0.03, 0.04]	**< .001**	0.965
Time(T2)*Emotional	0.08	[0.02, 0.13]	**0.008**		-0.09	[-0.12, -0.06]	**< .001**		-0.12	[-0.17, -0.06]	**< .001**		NA	NA	NA	
Time(T3)*Emotional	0.10	[0.05, 0.16]	**< .001**		-0.16	[-0.19, -0.12]	**< .001**		-0.18	[-0.24, -0.12]	**< .001**		NA	NA	NA	
Cognitive	-0.34	[-0.36, -0.31]	**< .001**	0.072	0.39	[0.37, 0.41]	**< .001**	**< .001**	0.46	[0.43, 0.49]	**< .001**	**0.005**	0.02	[0.02, 0.02]	**< .001**	0.747
Time(T2)*Cognitive	NA	NA	NA		-0.04	[-0.06, -0.02]	**< .001**		-0.03	[-0.06, 0.01]	**0.118**		NA	NA	NA	
Time(T3)*Cognitive	NA	NA	NA		-0.07	[-0.08, -0.05]	**< .001**		-0.05	[-0.09, -0.02]	**0.001**		NA	NA	NA	
**Executive function skills**																
Inhibition	-0.85	[-0.94, -0.75]	**< .001**	**0.002**	1.15	[1.09, 1.20]	**< .001**	**< .001**	1.15	[1.06, 1.25]	**< .001**	**0.011**	0.05	[0.05, 0.06]	**< .001**	0.149
Time(T2)*Inhibition	0.10	[0.00, 0.19]	**0.042**		-0.11	[-0.17, -0.06]	**< .001**		-0.08	[-0.18, 0.01]	0.091		NA	NA	NA	
Time(T3)*Inhibition	0.17	[0.07, 0.26]	**< .001**		-0.21	[-0.26, -0.15]	**< .001**		-0.15	[-0.24, -0.05]	**0.003**		NA	NA	NA	
Self-Monitoring	-1.29	[-1.42, -1.16]	**< .001**	**0.008**	1.49	[1.40, 1.58]	**< .001**	**< .001**	1.71	[1.57, 1.85]	**< .001**	**< .001**	0.07	[0.06, 0.08]	**< .001**	0.415
Time(T2)*Monitoring	0.17	[0.04, 0.30]	**0.013**		-0.15	[-0.23, -0.07]	**< .001**		-0.13	[-0.26, 0.01]	0.062		NA	NA	NA	
Time(T3)*Monitoring	0.20	[0.06, 0.33]	**0.005**		-0.27	[-0.35, -0.19]	**< .001**		-0.26	[-0.40, -0.12]	**< .001**		NA	NA	NA	
Shifting	-1.05	[-1.14, -0.96]	**< .001**	**0.004**	1.12	[1.06, 1.18]	**< .001**	**< .001**	1.38	[1.28, 1.48]	**< .001**	**< .001**	0.05	[0.05, 0.06]	**< .001**	0.846
Time(T2)*Shifting	0.13	[0.04, 0.23]	**0.005**		-0.12	[-0.17, -0.06]	**< .001**		-0.13	[-0.23, -0.04]	**0.006**		NA	NA	NA	
Time(T3)*Shifting	0.14	[0.05, 0.24]	**0.004**		-0.22	[-0.27, -0.16]	**< .001**		-0.24	[-0.34, -0.14]	**< .001**		NA	NA	NA	
Emotional Control	-1.26	[-1.37, -1.14]	**< .001**	**0.002**	1.50	[1.43, 1.57]	**< .001**	**< .001**	1.95	[1.83, 2.07]	**< .001**	**< .001**	0.08	[0.08, 0.09]	**< .001**	0.769
Time(T2)*EmoContr	0.11	[-0.01, 0.23]	0.061		-0.21	[-0.28, -0.14]	**< .001**		-0.29	[-0.41, -0.17]	**< .001**		NA	NA	NA	
Time(T3)*EmoContr	0.22	[0.10, 0.34]	**< .001**		-0.34	[-0.41, -0.26]	**< .001**		-0.40	[-0.52, -0.28]	**< .001**		NA	NA	NA	
Task Completion	-0.94	[-1.03, -0.86]	**< .001**	0.054	1.09	[1.03, 1.16]	**< .001**	**< .001**	1.27	[1.17, 1.38]	**< .001**	**0.018**	0.05	[0.05, 0.06]	**< .001**	0.735
Time(T2)*Task	NA	NA	NA		-0.11	[-0.17, -0.05]	**< .001**		-0.06	[-0.16, 0.04]	0.222		NA	NA	NA	
Time(T3)*Task	NA	NA	NA		-0.18	[-0.24, -0.12]	**< .001**		-0.15	[-0.25, -0.05]	**0.005**		NA	NA	NA	
Working Memory	-0.94	[-1.02, -0.85]	**< .001**	**0.030**	1.06	[1.00, 1.12]	**< .001**	**< .001**	1.21	[1.12, 1.31]	**< .001**	**0.007**	0.05	[0.04, 0.06]	**< .001**	0.571
Time(T2)*WM	0.07	[-0.02, 0.16]	0.106		-0.11	[-0.17, -0.06]	**< .001**		-0.09	[-0.18, 0.00]	0.063		NA	NA	NA	
Time(T3)*WM	0.12	[0.03, 0.21]	**0.009**		-0.20	[-0.25, -0.14]	**< .001**		-0.15	[-0.24, -0.06]	**0.002**		NA	NA	NA	
Planning	-0.82	[-0.88, -0.76]	**< .001**	0.310	0.93	[0.88, 0.98]	**< .001**	**< .001**	1.13	[1.05, 1.21]	**< .001**	**0.005**	0.05	[0.04, 0.05]	**< .001**	0.905
Time(T2)*Planning	NA	NA	NA		-0.08	[-0.12, -0.03]	**0.001**		-0.05	[-0.13, 0.02]	0.172		NA	NA	NA	
Time(T3)*Planning	NA	NA	NA		-0.15	[-0.19, -0.10]	**< .001**		-0.13	[-0.21, -0.05]	**0.001**		NA	NA	NA	

Note: We present the unstandardized regression coefficients to show the actual change in the outcome measure associated with a one-unit change in the predictor. Executive function, the underpinning subdimensions and skills, were cluster-mean centered and treated as time-constant (i.e., analyzed only at T1). Please note that higher executive function scores mean greater difficulties. Students are nested within schools. LRT=likelihood ratio test to evaluate whether the time*predictor interaction effect needs to be included in the model. NA=only the main effect of time, but not the time*predictor interaction term, was included. T1 is the reference. [¥]To address convergence problems, this model was estimated with the "bobyqa" optimizer for a quadratic approximation and full maximum likelihood estimation.

.

Table S8. Multivariable analyses for girls, based on the three-level random intercept model.

	Well-Being			Social-Emotional-Behavioral Difficulties			Depression			Suicidality		
	B	95% CI	p	B	95% CI	p	B	95% CI	p	B	95% CI	p
**Model 1: Executive function (total [EF])**												
EF	-0.25	[-0.27, -0.24]	**< .001***	0.25	[0.24, 0.26]	**< .001***	0.37	[0.35, 0.38]	**< .001***	0.02	[0.02, 0.02]	**< .001***
Time(T2)*EF	0.03	[0.02, 0.05]	**< .001***	-0.03	[-0.03, -0.02]	**< .001***	-0.05	[-0.06, -0.03]	**< .001***	NA	NA	NA
Time(T3)*EF	0.04	[0.02, 0.05]	**< .001***	-0.04	[-0.05, -0.03]	**< .001***	-0.06	[-0.07, -0.04]	**< .001***	NA	NA	NA
**Model 2: Self-regulation**												
Behavioral	-0.06	[-0.14, 0.02]	0.156	0.27	[0.22, 0.32]	**< .001***	0.07	[-0.03, 0.16]	0.189	0.02	[0.01, 0.03]	**< .001***
Time(T2)*Behavioral	0.01	[-0.07, 0.10]	0.779	-0.04	[-0.09, 0.01]	0.131	-0.00	[-0.11, 0.09]	0.921	NA	NA	NA
Time(T3)*Behavioral	0.00	[-0.08, 0.09]	0.875	-0.05	[-0.10, 0.00]	**0.038**	-0.04	[-0.14, 0.06]	0.472	NA	NA	NA
Emotional	-0.59	[-0.66, -0.51]	**< .001***	0.47	[0.42, 0.51]	**< .001***	1.10	[1.00, 1.19]	**< .001***	0.04	[0.03, 0.05]	**< .001***
Time(T2)*Emotional	0.15	[0.07, 0.23]	**< .001***	-0.16	[-0.21, -0.12]	**< .001***	-0.30	[-0.40, -0.21]	**< .001***	NA	NA	NA
Time(T3)*Emotional	0.16	[0.08, 0.24]	**< .001***	-0.18	[-0.22, -0.13]	**< .001***	-0.31	[-0.41, -0.22]	**< .001***	NA	NA	NA
Cognitive	-0.16	[-0.21, -0.11]	**< .001***	0.11	[0.08, 0.14]	**< .001***	0.10	[0.04, 0.16]	**< .001***	0.00	[0.00, 0.01]	0.245
Time(T2)*Cognitive	-0.02	[-0.07, 0.03]	0.418	0.06	[0.03, 0.09]	**< .001***	0.08	[0.01, 0.14]	**0.015***	0.00	[0.00, 0.01]	**0.049**
Time(T3)*Cognitive	-0.01	[-0.07, 0.04]	0.592	0.04	[0.01, 0.07]	**0.004***	0.08	[0.02, 0.14]	**0.012***	0.00	[0.00, 0.01]	**0.036**
**Model 3: Executive function skills**												
Inhibition	0.10	[-0.03, 0.22]	0.142	0.33	[0.25, 0.41]	**< .001***	-0.12	[-0.27, 0.03]	0.129	0.01	[0.00, 0.02]	0.146
Time(T2)*Inhibition	-0.03	[-0.16, 0.10]	0.659	-0.04	[-0.12, 0.04]	0.285	0.03	[-0.13, 0.19]	0.739	NA	NA	NA
Time(T3)*Inhibition	-0.09	[-0.23, 0.04]	0.186	-0.05	[-0.13, 0.02]	0.178	0.04	[-0.12, 0.20]	0.652	NA	NA	NA
Self-Monitoring	-0.23	[-0.41, -0.06]	**0.010***	0.08	[-0.02, 0.19]	0.126	0.20	[-0.01, 0.41]	0.066	0.03	[0.01, 0.04]	**0.005***
Time(T2)*Monitoring	0.06	[-0.12, 0.24]	0.515	-0.01	[-0.12, 0.09]	0.819	-0.04	[-0.26, 0.18]	0.736	NA	NA	NA
Time(T3)*Monitoring	0.15	[-0.03, 0.34]	0.110	-0.02	[-0.13, 0.08]	0.663	-0.11	[-0.33, 0.12]	0.350	NA	NA	NA
Shifting	-0.30	[-0.43, -0.16]	**< .001***	0.17	[0.09, 0.25]	**< .001***	0.40	[0.24, 0.56]	**< .001***	-0.01	[-0.02, 0.01]	0.452
Time(T2)*Shifting	0.06	[-0.08, 0.20]	0.420	-0.07	[-0.15, 0.01]	0.090	-0.19	[-0.36, -0.03]	**0.024**	NA	NA	NA
Time(T3)*Shifting	0.08	[-0.06, 0.22]	0.270	-0.10	[-0.18, -0.02]	**0.019***	-0.17	[-0.34, 0.00]	**0.044**	NA	NA	NA
Emotional Control	-0.82	[-0.94, -0.70]	**< .001***	0.73	[0.66, 0.80]	**< .001***	1.67	[1.53, 1.81]	**< .001***	0.08	[0.06, 0.09]	**< .001***
Time(T2)*EmoContr	0.22	[0.10, 0.34]	**< .001***	-0.24	[-0.32, -0.17]	**< .001***	-0.39	[-0.53, -0.24]	**< .001***	NA	NA	NA
Time(T3)*EmoContr	0.21	[0.09, 0.34]	**< .001***	-0.25	[-0.32, -0.18]	**< .001***	-0.42	[-0.57, -0.28]	**< .001***	NA	NA	NA
Task Completion	-0.07	[-0.22, 0.07]	0.317	0.04	[-0.05, 0.12]	0.427	-0.04	[-0.21, 0.14]	0.688	-0.00	[-0.02, 0.01]	0.574
Time(T2)*Task	-0.00	[-0.15, 0.15]	0.994	0.03	[-0.05, 0.12]	0.441	0.04	[-0.14, 0.22]	0.692	NA	NA	NA
Time(T3)*Task	0.02	[-0.14, 0.17]	0.836	0.08	[-0.01, 0.17]	0.084	0.19	[0.00, 0.37]	**0.045**	NA	NA	NA
Working Memory	-0.22	[-0.34, -0.09]	**< .001***	0.26	[0.18, 0.34]	**< .001***	0.22	[0.07, 0.37]	**0.005***	0.00	[-0.01, 0.02]	0.470
Time(T2)*WM	0.02	[-0.12, 0.15]	0.822	0.04	[-0.04, 0.12]	0.298	0.15	[-0.01, 0.31]	0.059	0.01	[0.00, 0.02]	**0.015***
Time(T3)*WM	-0.04	[-0.18, 0.09]	0.511	0.02	[-0.05, 0.10]	0.552	0.16	[0.00, 0.32]	**0.046**	0.01	[0.00, 0.02]	**0.008***
Planning	-0.28	[-0.41, -0.16]	**< .001***	0.16	[0.08, 0.23]	**< .001***	0.37	[0.23, 0.52]	**< .001***	0.02	[0.01, 0.04]	**< .001***
Time(T2)*Planning	-0.03	[-0.16, 0.09]	0.598	0.06	[-0.02, 0.13]	0.127	-0.00	[-0.15, 0.15]	0.987	NA	NA	NA
Time(T3)*Planning	0.02	[-0.11, 0.15]	0.794	0.00	[-0.07, 0.08]	0.928	-0.13	[-0.29, 0.02]	0.093	NA	NA	NA

Note: We present the unstandardized regression coefficients to show the actual change in the outcome measure associated with a one-unit change in the predictor. Executive function, the underpinning subdimensions and skills, were cluster-mean centered and treated as time-constant (i.e., analyzed only at T1). Please note that higher executive function scores mean greater difficulties. Models were adjusted for cohort, allocation, age (cluster centered), and ethnicity, all measured at baseline. Students are nested within schools. NA=only the main effect of time, but not the time*predictor interaction term, was included. T1 is the reference. [¥]To address convergence problems, this model was estimated with the "bobyqa" optimizer for a quadratic approximation and full maximum likelihood estimation. *Significant (p<.05) after adjustment for multiple comparisons.

Table S9. Multivariable analyses for boys, based on the three-level random intercept model.

	Well-being			Social-Emotional-Behavioral Difficulties			Depression			Suicidality		
	B	95% CI	p	B	95% CI	p	B	95% CI	p	B	95% CI	p
**Model 1: Executive function (total [EF])**												
EF	-0.19	[-0.21, -0.17]	**< .001***	0.22	[0.21, 0.23]	**< .001***	0.25	[0.24, 0.27]	**< .001***	0.01	[0.01, 0.01]	**< .001***
Time(T2)*EF	0.02	[0.00, 0.03]	**0.027**	-0.02	[-0.03, -0.01]	**< .001***	-0.02	[-0.04, 0.00]	**0.014***	NA	NA	NA
Time(T3)*EF	0.03	[0.01, 0.04]	**0.002***	-0.04	[-0.05, -0.03]	**< .001***	-0.04	[-0.05, -0.02]	**< .001***	NA	NA	NA
**Model 2: Self-regulation**												
Behavioral	0.05	[-0.05, 0.15]	0.295	0.29	[0.23, 0.35]	**< .001***	-0.02	[-0.13, 0.08]	0.686	0.00	[0.00, 0.01]	0.096
Time(T2)*Behavioral	0.02	[-0.07, 0.11]	0.655	-0.02	[-0.08, 0.05]	0.601	0.03	[-0.08, 0.13]	0.644	NA	NA	NA
Time(T3)*Behavioral	0.06	[-0.04, 0.15]	0.249	-0.05	[-0.11, 0.02]	0.142	0.02	[-0.09, 0.13]	0.730	NA	NA	NA
Emotional	-0.41	[-0.50, -0.31]	**< .001***	0.42	[0.36, 0.49]	**< .001***	0.82	[0.72, 0.93]	**< .001***	0.03	[0.02, 0.03]	**< .001***
Time(T2)*Emotional	0.06	[-0.03, 0.15]	0.166	-0.11	[-0.17, -0.05]	**< .001***	-0.26	[-0.37, -0.15]	**< .001***	NA	NA	NA
Time(T3)*Emotional	0.06	[-0.03, 0.15]	0.180	-0.16	[-0.23, -0.10]	**< .001***	-0.32	[-0.43, -0.21]	**< .001***	NA	NA	NA
Cognitive	-0.21	[-0.26, -0.15]	**< .001***	0.07	[0.04, 0.11]	**< .001***	0.10	[0.04, 0.16]	**0.002***	0.00	[0.00, 0.01]	**0.024**
Time(T2)*Cognitive	NA	NA	NA	0.02	[-0.02, 0.06]	0.293	0.08	[0.01, 0.15]	**0.017***	NA	NA	NA
Time(T3)*Cognitive	NA	NA	NA	0.03	[-0.01, 0.07]	0.134	0.08	[0.01, 0.15]	**0.018***	NA	NA	NA
**Model 3: Executive function skills**												
Inhibition	0.16	[0.00, 0.31]	0.051	0.44	[0.35, 0.54]	**< .001***	-0.14	[-0.30, 0.02]	0.089	0.00	[-0.01, 0.01]	0.848
Time(T2)*Inhibition	0.01	[-0.15, 0.18]	0.866	-0.03	[-0.13, 0.07]	0.556	0.06	[-0.11, 0.23]	0.492	NA	NA	NA
Time(T3)*Inhibition	0.12	[-0.04, 0.29]	0.149	-0.07	[-0.17, 0.03]	0.179	0.11	[-0.06, 0.28]	0.218	NA	NA	NA
Self-Monitoring	-0.11	[-0.33, 0.11]	0.340	-0.01	[-0.14, 0.13]	0.908	0.09	[-0.14, 0.32]	0.430	0.01	[-0.01, 0.03]	0.214
Time(T2)*Monitoring	0.07	[-0.15, 0.30]	0.518	0.00	[-0.13, 0.15]	0.921	-0.02	[-0.25, 0.22]	0.885	NA	NA	NA
Time(T3)*Monitoring	-0.02	[-0.25, 0.21]	0.881	-0.00	[-0.14, 0.14]	0.981	-0.11	[-0.34, 0.13]	0.384	NA	NA	NA
Shifting	-0.29	[-0.47, -0.12]	**< .001***	0.13	[0.02, 0.24]	**0.019**	0.29	[0.11, 0.47]	**0.002***	-0.01	[-0.03, 0.00]	0.057
Time(T2)*Shifting	0.15	[-0.02, 0.32]	0.078	-0.02	[-0.13, 0.09]	0.731	-0.11	[-0.30, 0.08]	0.255	NA	NA	NA
Time(T3)*Shifting	-0.00	[-0.18, 0.17]	0.969	-0.07	[-0.18, 0.04]	0.209	-0.17	[-0.37, 0.02]	0.073	NA	NA	NA
Emotional Control	-0.51	[-0.68, -0.34]	**< .001***	0.71	[0.61, 0.82]	**< .001***	1.30	[1.13, 1.47]	**< .001***	0.06	[0.05, 0.07]	**< .001***
Time(T2)*EmoContr	-0.00	[-0.17, 0.17]	0.992	-0.20	[-0.30, -0.09]	**< .001***	-0.40	[-0.58, -0.22]	**< .001***	NA	NA	NA
Time(T3)*EmoContr	0.17	[-0.01, 0.34]	0.063	-0.25	[-0.36, -0.15]	**< .001***	-0.44	[-0.62, -0.26]	**< .001***	NA	NA	NA
Task Completion	-0.07	[-0.21, 0.08]	0.392	0.03	[-0.08, 0.14]	0.603	-0.04	[-0.23, 0.14]	0.644	0.00	[-0.01, 0.01]	0.974
Time(T2)*Task	NA	NA	NA	-0.01	[-0.13, 0.10]	0.827	0.08	[-0.12, 0.27]	0.435	NA	NA	NA
Time(T3)*Task	NA	NA	NA	0.04	[-0.08, 0.15]	0.542	0.08	[-0.12, 0.27]	0.450	NA	NA	NA
Working Memory	-0.12	[-0.28, 0.04]	0.155	0.14	[0.03, 0.24]	**0.009***	0.10	[-0.07, 0.27]	0.252	0.00	[-0.01, 0.01]	0.922
Time(T2)*WM	-0.07	[-0.23, 0.08]	0.346	-0.03	[-0.13, 0.08]	0.628	-0.02	[-0.19, 0.16]	0.862	NA	NA	NA
Time(T3)*WM	-0.03	[-0.18, 0.13]	0.728	-0.06	[-0.16, 0.05]	0.308	0.04	[-0.14, 0.22]	0.641	NA	NA	NA
Planning	-0.41	[-0.54, -0.28]	**< .001***	0.17	[0.07, 0.26]	**< .001***	0.41	[0.25, 0.57]	**< .001***	0.03	[0.02, 0.04]	**< .001***
Time(T2)*Planning	NA	NA	NA	0.06	[-0.04, 0.15]	0.254	0.11	[-0.05, 0.28]	0.185	NA	NA	NA
Time(T3)*Planning	NA	NA	NA	0.07	[-0.03, 0.17]	0.194	0.06	[-0.10, 0.23]	0.458	NA	NA	NA

Note: We present the unstandardized regression coefficients to show the actual change in the outcome measure associated with a one-unit change in the predictor. Executive function, the underpinning subdimensions and skills, were cluster-mean centered and treated as time-constant (i.e., analyzed only at T1). Please note that higher executive function scores mean greater difficulties. Models were adjusted for cohort, allocation, age (cluster centered), and ethnicity, all measured at baseline. Students are nested within schools. NA=only the main effect of time, but not the time*predictor interaction term, was included. T1 is the reference. [¥]To address convergence problems, this model was estimated with the "bobyqa" optimizer for a quadratic approximation and full maximum likelihood estimation. *Significant ($p<.05$) after adjustment for multiple comparisons.

Table S10. Adjusted regression coefficients for the relationship between executive function skills at T1 and mental health outcomes (T1 to T3) by gender, based on the three-level random intercept model.

Well-being (range: 14-70)

Whole sample

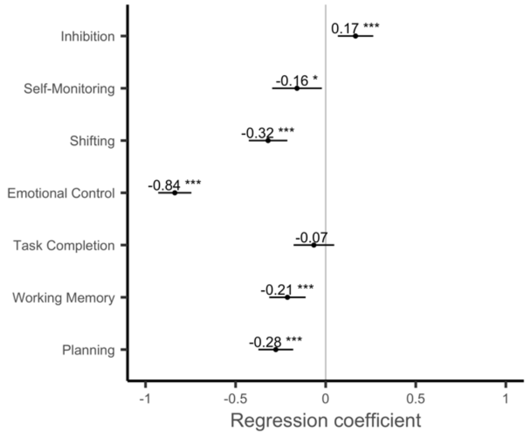

Girls

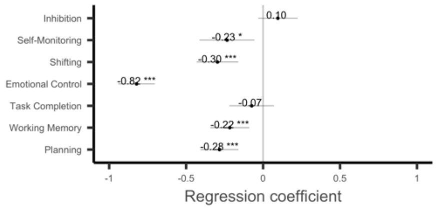

Boys

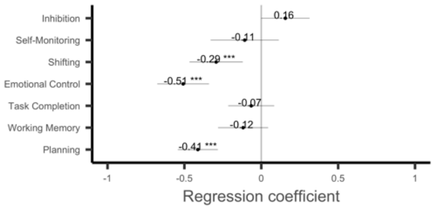

Social-Emotional-Behavioral Difficulties (range: 0-40)

Whole sample

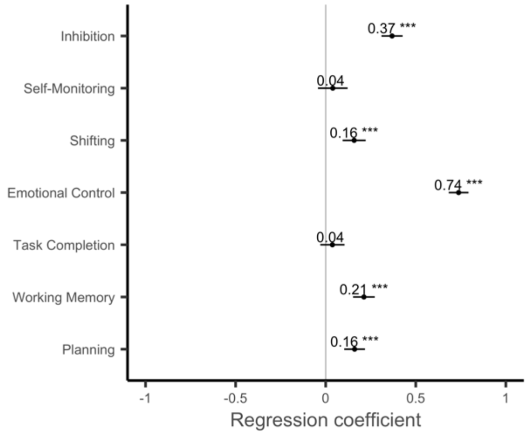

Girls

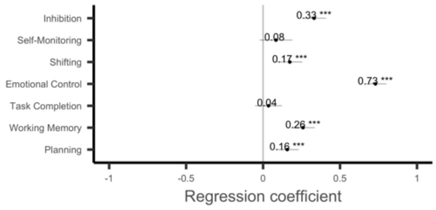

Boys

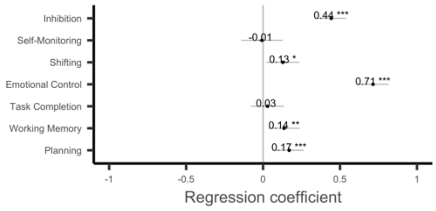

Depression (range: 0-60)

Whole sample

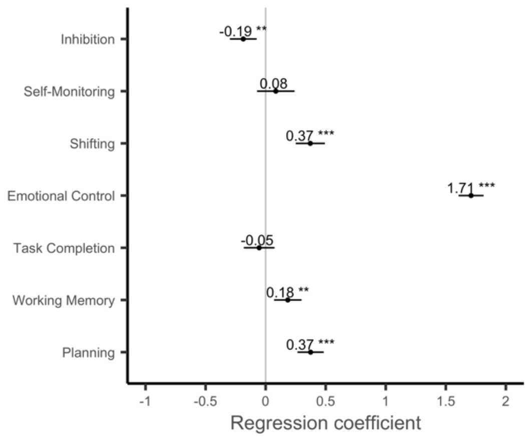

Girls

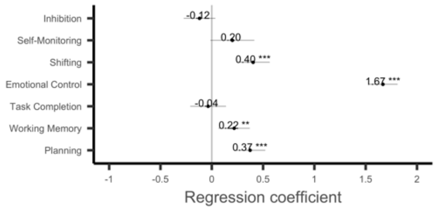

Boys

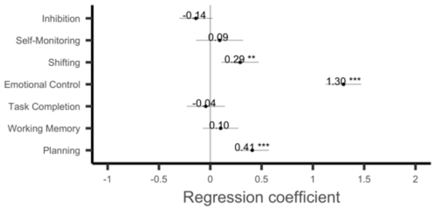

Suicidality (range: 0-3)

Whole sample

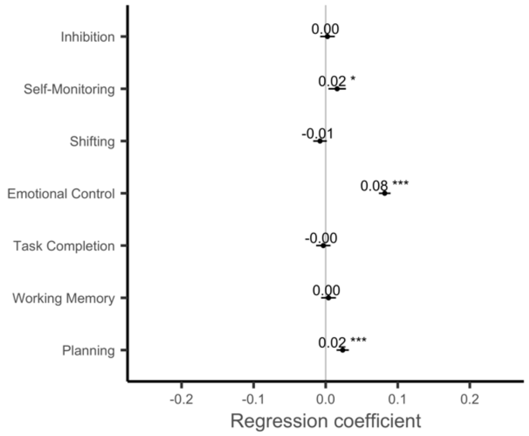

Girls

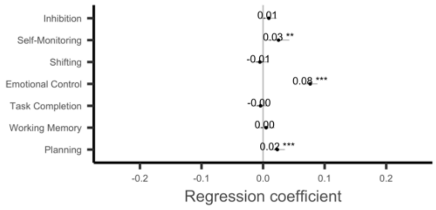

Boys

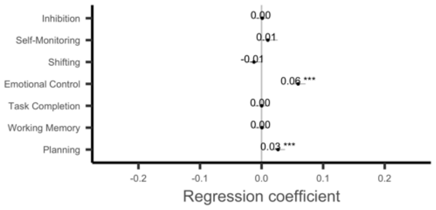

We present the rounded, unstandardized regression coefficients. The unstandardized regression coefficients represent the actual change in the outcome measure associated with a one-unit change in the respective executive function skill, while controlling for all other executive function skills, cohort, allocation, age (cluster centered, and ethnicity; see also **Tables S7-S8**). Due to the different score ranges of our executive function skills and mental health outcomes, these coefficients are not directly comparable and should therefore only be interpreted based on the width of the 95% confidence interval and whether it crosses zero, as an indication of statistical significance and robustness of the reported effects. Higher executive function scores mean greater difficulties.

References for the supplements

Bates, D., Machler, M., Bolker, B.M., Walker, S.C. (2015). Fitting Linear Mixed-Effects Models Using lme4. *Journal of Statistical Software, 67,* 1–48.

Brunson, J.C., & Read, Q.D. (2020). *ggalluvial: Alluvial Plots in 'ggplot2'.* R package version 0.12.3. http://corybrunson.github.io/ggalluvial/

Brunson, J.C. (2020). ggalluvial: Layered Grammar for Alluvial Plots. *Journal of Open Source Software, 5,* 2017. doi: 10.21105/joss.02017.

Clarke, A., Friede, T., Putz, R., Ashdown, J., Martin, S., Blake, A., Adi, Y., Parkinson, J., Flynn, P., Platt, S., Stewart-Brown, S. (2011). Warwick-Edinburgh Mental Well-being Scale (WEMWBS): Validated for teenage school students in England and Scotland. A mixed methods assessment. *BMC Public Health, 11,* 487. doi: 10.1186/1471-2458-11-487.

Dierker, L.C., Albano, A.M., Clarke, G.N., Heimberg, R.G., Kendall, P.C., Merikangas, K.R., Lewinsohn, P.M., Offord, D.R., Kessler, R., & Kupfer, D.J. (2001) Screening for anxiety and depression in early adolescence. *Journal of the American Academy of Child and Adolescent Psychiatry, 40,* 929-936. doi: 10.1097/00004583-200108000-00015.

Fox, J., & Weisberg, S. (2019). *An R Companion to Applied Regression, Third Edition.* Thousand Oaks, CA: Sage.

Garbuszus, J.M., Pfaff, B. (2021). *readspss: Importing and Exporting SPSS Files*. R package version 0.14.

Garnier, S., Ross, N., Rudis, R., Camargo, A.P, Sciaini, M., & Scherer, C. (2021). *viridis - Colorblind-Friendly Color Maps for R.* R package version 0.6.2.

Garrison, C.Z., Addy, C.L., Jackson, K.L., McKeown, R.E., & Waller, J.L. (1991). The CES-D as a Screen for Depression and Other Psychiatric-Disorders in Adolescents. *Journal of the American Academy of Child and Adolescent Psychiatry, 30,* 636-641. doi: 10.1097/00004583-199107000-00017.

Gioia, G.A., Isquith, P.K., Guy, S.C., & Kenworthy, L. (2015). *Behavior Rating Inventory of Executive Function, Second Edition (BRIEF-2) - Professional Manual.* Lutz, Florida (USA): Psychological Assessment Resources Inc.

Goodman, R. (2001). Psychometric properties of the strengths and difficulties questionnaire. *Journal of the American Academy of Child & Adolescent Psychiatry, 40,* 1337–1345.

Harrell, F.E. (2021). *Hmisc: Harrell Miscellaneous.* R package version 4.6-0.

Kirtley, O.J., Hussey, I., & Marzano, L. (2021). Exposure to and experience of self-harm and self-harm related content: An exploratory network analysis. *Psychiatry Research, 295,* 113572. doi: 10.1016/j.psychres.2020.113572.

Kidger, J., Heron, J., Lewis, G., Evans, J., & Gunnell, D. (2012) Adolescent self-harm and suicidal thoughts in the ALSPAC cohort: a self-report survey in England. *BMC Psychiatry, 12,* e69.

Kuyken, W., Nuthall, E., Byford, S., … & MYRIAD Team (2017). The effectiveness and cost-effectiveness of a mindfulness training programme in schools compared with normal school provision (MYRIAD): study protocol for a randomised controlled trial. *Trials,* 18, e194. 10.1186/s13063-017-1917-4.

Lüdecke, D. (2021). *sjPlot: Data Visualization for Statistics in Social Science*. R package version 2.8.9.

Lüdecke, D., Ben-Shachar, M., Patil, I., Makowski, D. (2020). Extracting, Computing and Exploring the Parameters of Statistical Models using R. *Journal of Open Source Software, 5,* 2445.

Lüdecke, D., Ben-Shachar, M., Patil, I., Waggoner, P., & Makowski, D. (2021). performance: An R Package for Assessment, Comparison and Testing of Statistical Models. *Journal of Open Source Software, 6,* 3139.

R Core Team. (2019). *R: A language and environment for statistical computing. R version 3.6.2 – "Dark and Stormy Night"*. Vienna, Austria: R Foundation for Statistical Computing.

Radloff, L.S. (1977). The CES-D Scale: A self-report depression scale for research in the general population. *Applied Psychological Measurement, 1,* 385–401.

Radloff, L.S. (1991). The use of the Center for Epidemiologic Studies Depression Scale in adolescents and young adults. *Journal of Youth and Adolescence, 20,* 149–66.

Revelle, W. (2021). *psych: Procedures for Personality and Psychological Research*. R package version 2.1.9. Evanston, Illinois, USA: Northwestern University.

Roberts, R.E., Andrews, J.A., Lewinsohn, P.M., & Hops, H. (1990). Assessment of depression in adolescents using the Center for Epidemiologic Studies Depression Scale. *Psychological Assessment: A Journal of Consulting and Clinical Psychology, 2*, 122–128. doi: 10.1037/1040-3590.2.2.122.

Tennant, R., Hiller, L., Fishwick, R., Platt, S., Joseph, S., Weich, S., Parkinson, J., Secker, J., & Stewart Brown, S. (2007). The Warwick-Edinburgh mental well-being scale (WEMWBS): development and UK validation. *Health and Quality of Life Outcomes, 5,* 63. doi: 10.1186/1477-7525-5-63.

Warwick Medical School (2021). *Collect, score, analyse and interpret WEMWBS.* [accessed 09.11.2022]; Available from: https://warwick.ac.uk/fac/sci/med/research/platform/wemwbs/using/howto/.

Wickham, H. (2016). *ggplot2: Elegant Graphics for Data Analysis*. New York, USA: Springer-Verlag.

Wickham, H., & Girlich, M. (2022). *tidyr: Tidy Messy Data*. R package version 1.2.0.

Wickham, H., François, R., Henry, L., & Müller, K. (2022). *dplyr: A Grammar of Data Manipulation.* R package version 1.0.8.

Wickham, H., Averick, M., Bryan, J., Chang, W., D'Agostino McGowan, L., François, R., Grolemund, G., Hayes, A., Henry, L., Hester, J., Kuhn, M., Lin Pedersen, T., Miller, E., Milton Bache, S., Müller, K., Ooms, J., Robinson, D., Paige Seidel, D., Spinu, V., Takahashi, K., Vaughan, D., Wilke, C., Woo, K., & Yutani, H. (2019). Welcome to the tidyverse. *Journal of Open Source Software, 4,* 1686. doi: 10.21105/joss.01686.
Yanagida, T. (2021). *misty: Miscellaneous Functions 'T. Yanagida'.* R package version 0.4.3.
Youth in Mind. (2016). Scoring the SDQ. Available from: https://sdqinfo.org/py/sdqinfo/c0.py.
